# Insights into the Evolution of Neoteny from the Genome of the Asian Icefish *Protosalanx chinensis*

**DOI:** 10.1016/j.isci.2020.101267

**Published:** 2020-06-14

**Authors:** Jie Zhang, Jiwei Qi, Fanglei Shi, Huijuan Pan, Meng Liu, Ran Tian, Yuepan Geng, Huaying Li, Yujie Qu, Jinping Chen, Inge Seim, Ming Li

**Affiliations:** 1 Chinese Academy of Sciences Key Laboratory of Animal Ecology and Conservation Biology, Institute of Zoology, Beijing 100101, China; 2 University of Chinese Academy of Sciences, Beijing 100049, China; 3 School of Ecology and Nature Conservation, Beijing Forestry University, Beijing 100083, China; 4 Novogene Bioinformatics Institute, Beijing 100083, China; 5 Integrative Biology Laboratory, College of Life Sciences, Nanjing Normal University, Nanjing 210046, China; 6 Guangdong Key Laboratory of Animal Conservation and Resource, Utilization, Guangdong Public Laboratory of Wild Animal Conservation and Utilization, Guangdong Institute of Applied Biological Resources, Guangzhou 510260, China; 7 Comparative and Endocrine Biology Laboratory, Translational Research Institute-Institute of Health and Biomedical Innovation, School of Biomedical Sciences, Queensland University of Technology, Woolloongabba, QLD 4102, Australia; 8 Center for Excellence in Animal Evolution and Genetics, Chinese Academy of Sciences, Kunming 650223, China

**Keywords:** Biological Sciences, Evolutionary Biology, Genomics

## Abstract

Salangids, known as Asian icefishes, represent a peculiar radiation within the bony fish order Protacanthopterygii where adult fish retain larval characteristics such as transparent and miniaturized bodies and a cartilaginous endoskeleton into adulthood. Here, we report a *de novo* genome of *Protosalanx chinensis*, the most widely distributed salangid lineage. The *P. chinensis* genome assembly is more contiguous and complete than a previous assembly. We estimate that *P. chinensis*, salmons, trouts, and pikes diverged from a common ancestor 185 million years ago. A juxtaposition with other fish genomes revealed loss of the genes encoding ectodysplasin-A receptor (*EDAR*), SCPP1, and four *Hox* proteins and likely lack of canonical fibroblast growth factor 5 (*FGF5*) function. We also report genomic variations of *P. chinensis* possibly reflecting the immune system repertoire of a species with a larval phenotype in sexually mature individuals. The new Asian icefish reference genome provides a solid foundation for future studies.

## Highlights

• Generated chromosome-level genome of the Asian icefish *Protosalanx chinensis*
• Larval features present in adult *P. chinensis* (neoteny)
• Identified genes underlying icefish neoteny, including an adult cartilaginous skeleton
• Valuable resource for wet-lab and genome research on icefishes

## Graphical Abstract

**Figure:**
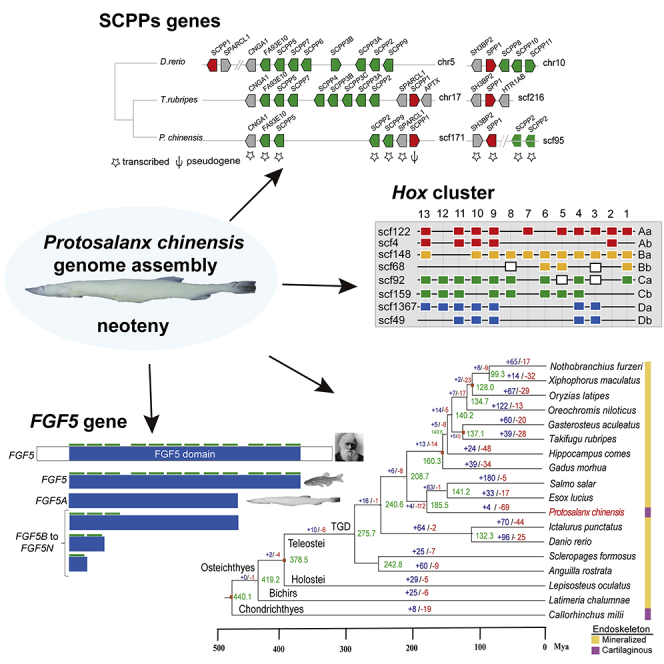

## Introduction

**Figure 1:**
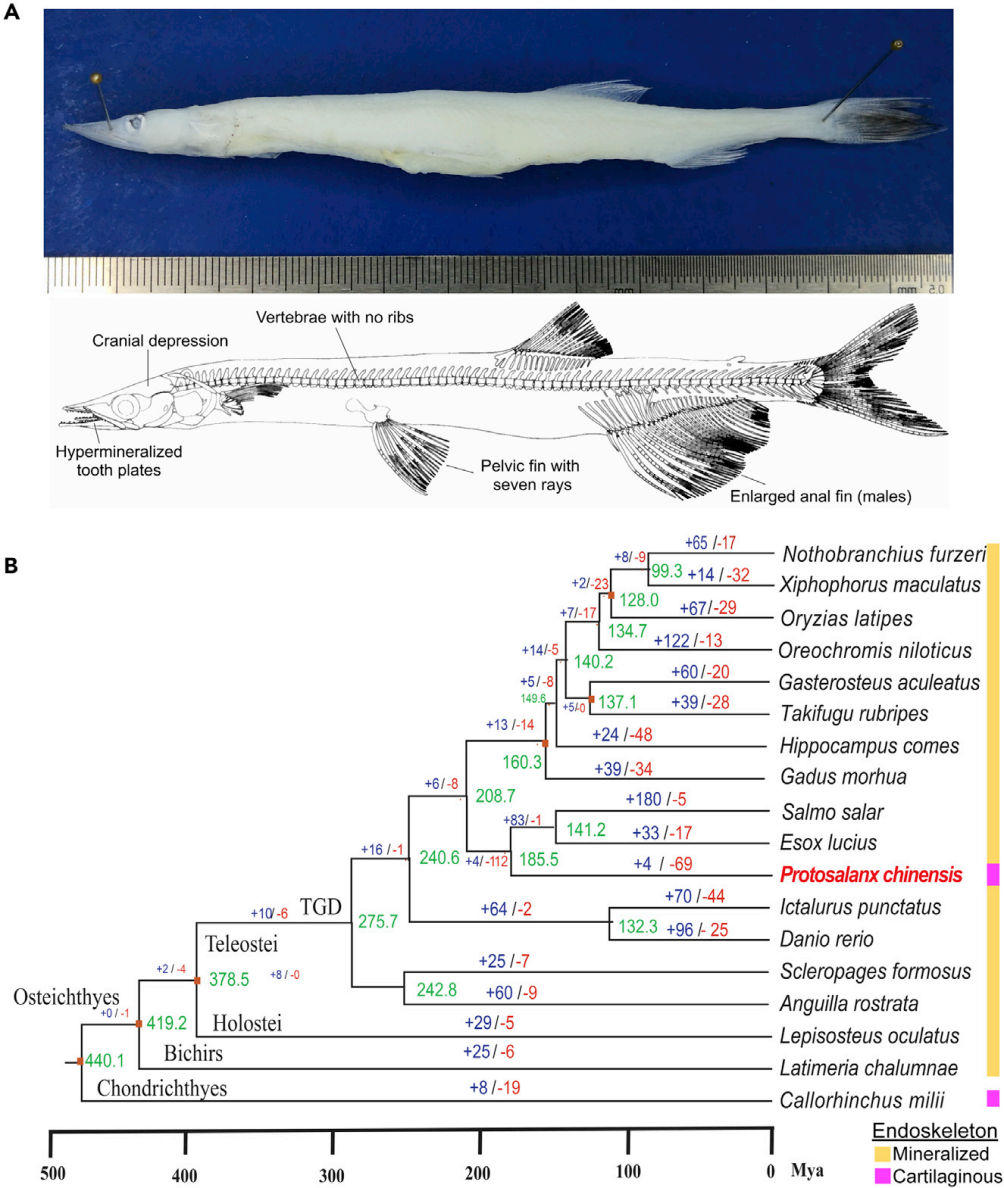
*P. chinensis* and a Phylogenetic Tree Showing Gene/Family Expansions/Contractions Analysis Compared with 17 Representative Fish Species (A) Top: an adult female *P. chinensis*, an Asian icefish, collected from Chaohu lake, Anhui province. Bottom: skeletal features of *P. chinensis*. In contrast to other bony fishes, and similar to distantly related cartilaginous fishes such as the elephant fish (*Callorhinchus milii*), *P. chinensis* cannot produce a mineralized endoskeleton (e.g., neurocranium, vertebrae) from larval cartilage precursors. Prominent exoskeletal features (dermal bones, such as fin rays and teeth) of *P. chinensis* are indicated. (B) Consensus phylogenetic tree of 18 teleost fishes. The tree was generated from 627 single-copy genes. The divergence times (million years ago; mya; shown in green) for all nodes were estimated based on the six red nodes with fossil records as calibration times and are marked in each node with error ranges. Gene family expansion events are marked in blue, and gene family contraction events in red. A gene duplication event at the base of teleosts (TGD) is indicated. The type of adult endoskeletal bone (cartilaginous or mineralized) is indicated in pink and yellow, respectively. See also Figures S1–S3 and Tables S1–S14.

Bony vertebrates develop a mineralized endoskeleton from a cartilaginous larval scaffold (endochondral ossification), whereas chondrichthyans (chimeras, sharks, skates, and rays) retain a cartilaginous endoskeleton throughout life (Hirasawa and Kuratani, 2015). The two bony fish lineages, lobe-finned fishes (lungfishes and coelacanths) and ray-finned fishes, are collectively also known as teleosts, derived from the Greek *teleios* + *osteon*, “complete bone” (Brazeau and Friedman, 2015). A peculiar radiation is observed in order Osmeriformes of Protacanthopterygii, a teleost superorder that also includes Esociformes (e.g., pikes) and Salmoniformes (e.g., salmons and trouts). Osmeriformes comprises the six-genera (∼17 species) family Salangidae of short-lived (lifespan ∼12 months; sexual maturity at 7 months of age), morphologically similar fishes endemic to East Asia and mainly distributed in China (Zhang et al., 2007). Members of Salangidae are known by many colloquial names (e.g., [Asian] icefishes, salangids, whitefishes, and noodlefishes) (Roberts, 1984). Adult Asian icefishes are small, transparent, and scaleless. They possess several larval features, including a cartilaginous endoskeleton and notochords throughout life (Nelson, 2006; Wu and Lin, 1965; Roberts, 1984). Such morphological and structural variants, or “developmental deviations,” are thought to be of great significance in fish (Wu and Lin, 1965). The present study aimed to explore the genetic features the enigmatic Asian icefish *Protosalanx chinensis* (Figure 1A). *P. chinensis* is one of the most ecologically plastic Asian icefish species (Roberts, 1984; Kang et al., 2015) and has the broadest geographical distribution (China, Korea, and Vietnam) (Kang et al., 2015). Freshwater stocks of *P. chinensis* occur in the inland lakes, reservoirs, and out-flowing rivers in China, whereas marine stocks are distributed in estuarine and coastal areas in east Asia (Zhang et al., 2007). We report an improved genome assembly and transcriptomes of *P. chinensis* and identify genomic variations that may be associated with its unusual features.

## Results and Discussion

### Genome Assembly and Annotation

By integrating PacBio technology, 10X Genomics linked-read sequencing, and Illumina short-read sequencing, we constructed a 484.1 Mb *P. chinensis* genome assembly with a contig N50 size of 103 kb and a scaffold N50 size of 5.1 Mb (Tables S1–S3, Figures S1A and S1B). Our long-read assembly is superior to a previously published assembly (genome size 525 Mb; contig N50 17.2 kb, scaffold N50 1.16 Mb) (Liu et al., 2017). The *P. chinensis* genome has an average GC content of 47.25%, higher than most other sequenced fishes (Table S4, Figures S1C and S1D). We mapped short-insert (250–500 bp) reads to the *P. chinensis* genome and found that 97.89% could be aligned (Table S5). The *P. chinensis* genome contains 31.97% repeat elements, the majority (10.77%) DNA transposons (Tables S6 and S7, Figure S1E). The assembly is of high quality, as >98% of *de novo* assembled transcripts could be mapped (Table S8). Moreover, CEGMA (Parra et al., 2007) and BUSCO (Waterhouse et al., 2017) completeness scores are 93% and 94%, respectively. The scores of the previous *P. chinensis* assembly (Liu et al., 2017) are 87% CEGMA and 85% BUSCO (Tables S9 and S10). We predicted 23,645 genes in the genome of *P. chinensis* by combining *ab initio* gene prediction, protein-based homology, and transcript-mapping strategies (Table S11, Figure S1F). The gene length in *P. chinensis* assembly averages 8,530 kb. Average exon and intron sizes are 0.17 and 0.91 kb, respectively, similar to other teleost fish (Table S12, Figure S2). Non-coding RNA annotation revealed 95 rRNA, 1,382 tRNA, 1,327 miRNA, and 1,025 snRNA genes (Table S13).

### Phylogenetic Placement of *P. chinensis*

*Protosalanx* is a monotypic genus, with reported species in addition to *P. chinensis* (e.g., *P. hyalocranius* and *P. anderssoni*) attributed to reports of *P. chinensis* under different species names and misclassification with other Asian icefishes (Roberts, 1984). *P. hyalocranius* and *P. chinensis* are synonyms (Zhang et al., 2007). Thus, the previously reported (Liu et al., 2017) Asian icefish genome of *P. hyalocranius* is the same species sequenced in our study. By examining 627 single-copy gene families from 18 sequenced fish genomes, we generated a phylogenetic tree in agreement with the fossil record (Benton et al., 2009; Bian et al., 2016; Schartl et al., 2013; Yang et al., 2016) (Figure 1B). The phylogeny places Osmeriformes (*P. chinensis*) as a sister order to Salmoniformes (*Salmo salar*) and Esociformes (*Esox lucius*). The divergence time between these orders was estimated to be about 185.5 million years ago (mya), the Jurassic period (Figures 1B and S3; Table S14).

### Molecular Basis for Bone and Scale Formation

**Figure 2:**
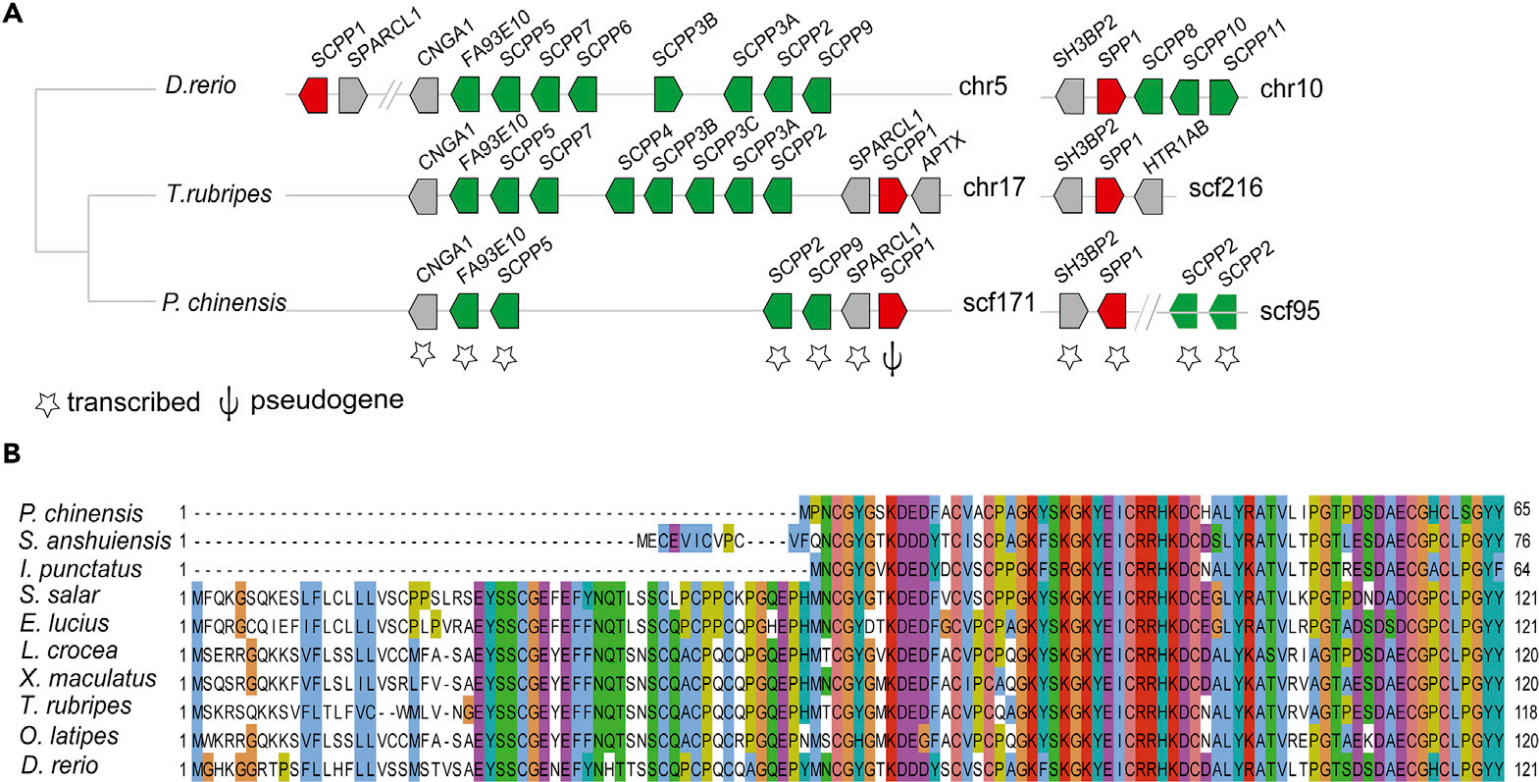
Loss of Bone Formation and Maintenance Genes in *P. chinensis* (A) Secretory calcium-binding phosphoproteins (SCPPs) genes in *P. chinensis*, *D. rerio* (zebrafish), and *T. rubripes* (fugu). *SPARCL1*, the ancestral SCPP gene, is shown in gray; P/Q-rich SCPP genes in green; acidic SCPP genes in red. In *P. chinensis*, *SCPP1* is a pseudogene (denoted by ψ). (B) *EDAR* (ectodysplasin-A receptor) protein sequences from ten fish species were aligned by MUSCLE. The coding sequence of the *EDAR* signal peptide region and parts of the extracellular region is lost in *P. chinensis*, the scaleless channel catfish *I. punctatus,* and the cavefish *S. anshuiensis*. See also Figures S4 and S5 and Tables S15 and S16.

*P. chinensis* belongs to the bony fishes (Osteichthyes), but its endoskeleton is composed of cartilage (Figure S4). In this sense, it is more similar to cartilaginous fishes such as sharks. In order to understand the genetic mechanism underlying the cartilaginous skeleton of *P. chinensis*, we next identified genes involved in bone formation and maintenance from a set of 166 genes (Venkatesh et al., 2014). We found that *P. chinensis* possesses intact orthologs for most genes involved in bone formation (Table S15). However, genes encoding matrix Gla protein (*MGP*) and osteocalcin (*BGLAP*, also known as bone Gla protein), and several secretory calcium-binding phosphoproteins (SCPPs) are absent in *P. chinensis*. *MGP* and osteocalcin are important regulators of calcium metabolism and skeletal development (Kawasaki et al., 2009). *MGP* and osteocalcin control bone mineralization, whereas SCPP genes have crucial functions in the mineralization of bone, dentin, enamel, and enameloid (Kawasaki and Weiss, 2003). The SCPP gene family arose by gene duplication from a common ancestor, *SPARCL1*. The SCPP family has two subclasses: acidic SCPPs and Pro/Gln (P/Q)-rich SCPPs (Kawasaki, 2009, 2011). Interrogation of the *P. chinensis* genome and transcriptomes showed that it has two acidic SCPP genes (*SPARCL1* and *SPP1*) and four P/Q-rich SCPP genes (*SCPP2*, *SCPP5*, *SCPP9*, and *FA93E10*) (Figure 2A). We found three copies of *SCPP2* (located on different scaffolds) in the *P. chinensis* genome. Further analysis of gene synteny—comparing *P. chinensis*, fugu (*T. rubripes*), and zebrafish (*D. rerio*) genomes—revealed that *SCPP4*, *SCPP3A*, *SCPP3B*, *SCPP3C*, *SCPP6*, *SCPP7*, and *SCPP8* are absent in the *P. chinensis* genome (Figure 2A). *SCPP1* sequence was identified in a highly syntenic region. However, only two of eight exons could be identified, and we could not detect its expression, indicating that it is a pseudogene*.* We found that two other species with cartilaginous skeletons, the elephant fish (*Callorhinchus milii*) (Venkatesh et al., 2014) and ocean sunfish (*Mola mola*) (Pan et al., 2016), have also lost SCPP genes. Loss of *SCPP1* or *SCPP5*, or both, may result in a scaleless phenotype in bony fishes. A scaleless three-spine stickleback (*Gasterosteus aculeatus*) has intact *SCPP1* but lacks *SCPP5*; a scaleless electric eel (*Electrophorus electricus*) (Gallant et al., 2014) has *SCPP5* but lost *SCPP1*; and a scaleless channel catfish (*Ictalurus punctatus*) has lost both *SCPP5* and *SCPP1* (Liu et al., 2016). The gene encoding the ectodysplasin-A receptor (*EDAR*) has deletions in the signal peptide and extracellular regions in *P. chinensis* (Figure 2B). Similarly, the gene lacks a signal peptide in the scaleless channel catfish (*Ictalurus punctatus*) (Kellogg, 1975) and cavefish (*Sinocyclocheilu asanshuiensis*) (Yang et al., 2016), and *EDAR* mutations in the gene exon region lead to complete scale loss in medaka (Kondo et al., 2001) and zebrafish (Harris et al., 2008). To complement the genome analysis, we profiled the *P. chinensis* transcriptome at four development stages (pharyngula, hatching, larva, and adult). The expression of most genes involved in ossification showed different levels among the stages. At the adult stage, highly expressed genes included proteoglycans and bone differentiation gene families (Figure S5; Table S16). Furthermore, although *SCPP2*, *SCPP5*, *SCPP9*, and *FA93E10* are intact in *P. chinensis*, their expression is low at all development stages. Taken together, we speculate that the cartilaginous skeleton of *P. chinensis* is manifested by various gene variations, with loss of *EDAR* and *SCPP1* emerging as the leading cause of complete scale loss.

### Loss of *Hox* Cluster *Ca* Genes in Distantly Related Fish Species with a Larval Phenotype

**Figure 3:**
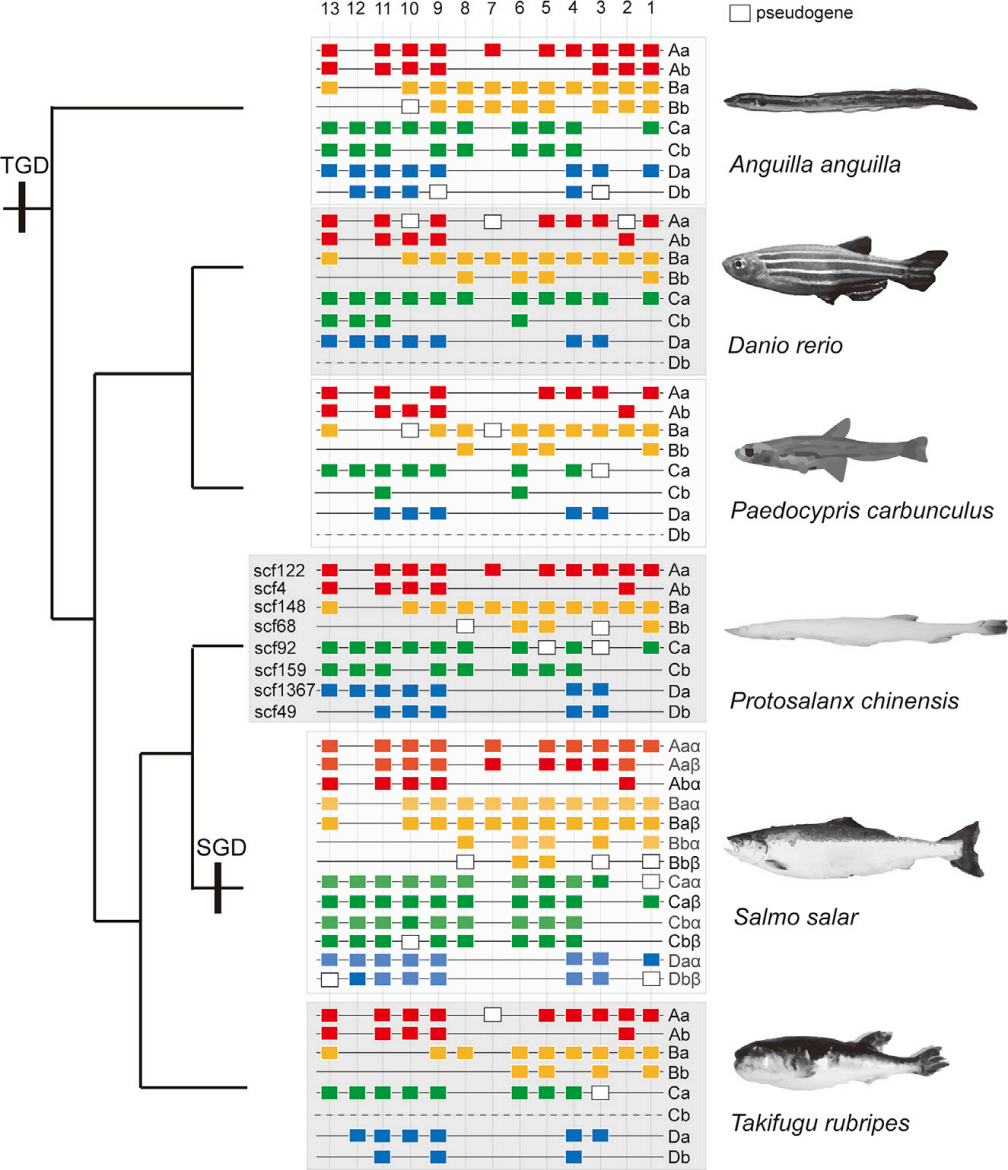
Phylogeny of *Hox* Gene Clusters of Six Teleosts Overview of the *Hox* gene clusters of *Anguilla anguilla* (European eel) (Minegishi et al., 2005), *Danio rerio* (zebrafish) (Bian et al., 2016), *Paedocypris carbunculus* (a dwarf minnow; common name yet to be assigned) (Malmstrom et al., 2018), *Protosalanx chinensis* (Asian icefish), *Salmo salar* (Atlantic salmon) (Mungpakdee et al., 2008), and *Takifugu rubripes* (fugu) (Bian et al., 2016). Each horizontal black line refers to a *Hox* cluster. Solid rectangles represent complete *HoxA* (red), *HoxB* (orange), *HoxC* (green), and *HoxD* (blue) genes, whereas hollow rectangles indicate pseudogenes or partial genes. Paralogs generated by TGD (a teleost whole-genome duplication event) are denoted “a” and “b,” whereas paralogs produced by lineage-specific SGD (a salmonid whole-genome duplication event) are denoted “α” and “β.” See also Table S17.

Homeobox (*Hox*) genes are highly conserved transcription factors organized into chromosomal clusters (Mallo, 2018). Extensive *Hox* gene loss (10 genes) was recently reported in two species of the Southeast Asian dwarf minnow genus *Paedocypris* (*P. carbunculus* and *P. micromegethes*) (Malmstrom et al., 2018). Similar to *P. chinensis*, adult fish in this genus retain larval features (developmental truncation). Four of 62 *Hox* genes are pseudogenized in *P. chinensis* (validated by PCR and Sanger sequencing) (Figure 3; Table S17). Two of the genes are also lost in *Paedocypris* (*HOXC5a* and *HOXC3a*) and are located in the same *Hox* cluster (*HOXCa*). These genes are expressed in the neural tube of fish embryos (Davis and Stellwag, 2010; le Pabic et al., 2009; Lyon et al., 2013). *P. chinensis* (Roberts, 1984) and *Paedocypris* (Kottelat et al., 2006) have a roofless skull (posterior portions are open throughout life); however, we are not aware of studies that have examined the effect of *HOXC5a* and *HOXC3a* loss on the development of the skeletal system of teleost fish. An assignment of function, and whether *HOXC5a* acts alone or in concert with *HOXC3a*, awaits further investigation.

### No Canonical Fibroblast Growth Factor 5 in *P. chinensis*

**Figure 4:**
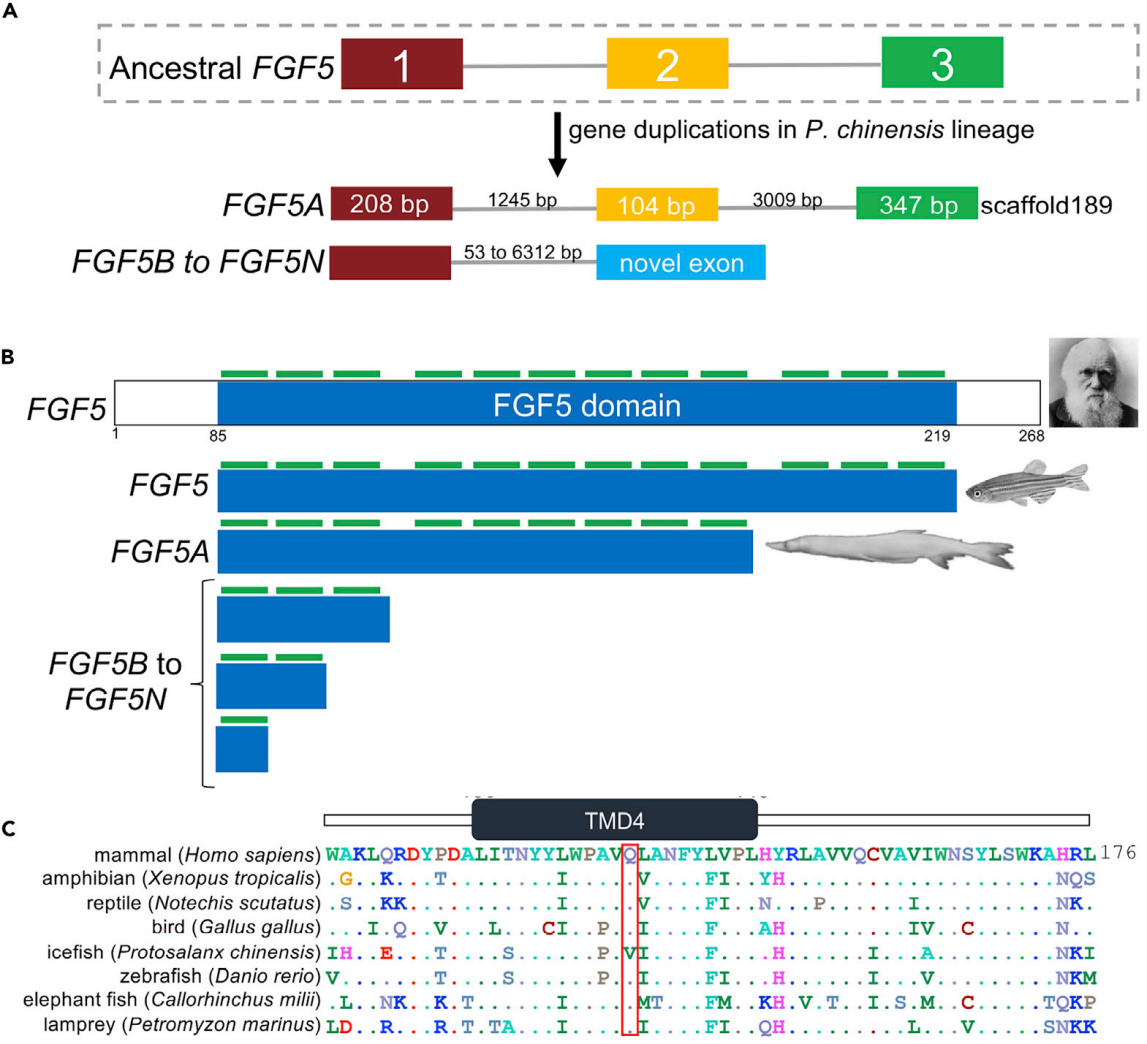
Fibroblast Growth Factor 5 Gene Copy Number Increase and a Unique Amino Acid Change of the Fish Pigmentation Gene Mitochondrial Inner Membrane Protein 17 in *P. chinensis* (A) One of the 86 gene families gained by *P. chinensis* include fibroblast growth factor 5-like genes. *P. chinensis* has two distinct *FGF5* gene types: a three-exon ortholog to *FGF5* of other fish species was found on scaffold189 (denoted *FGF5A*), whereas duplicated genes are part of a novel gene (*FGF5B* to *FGF5N*). (B) The domain structure of human, zebrafish, and *P. chinensis* FGF5-derived proteins is shown. The canonical FGF5 domain (shown in blue) has a highly conserved core region with 12 β strands (shown by green bars) within the core region of FGF family polypeptides. If translated, all *P. chinensis FGF5* genes (denoted *FGF5A* to *FGF5N*) would encode a C-terminally truncated FGF5 form. (C) Partial alignment of mitochondrial inner membrane protein 17 (*MPV17*) sequences in vertebrates. MPV17 transmembrane domain four is shaded in green. An amino acid change (Gln142Val) unique to *P. chinensis* is highlighted in red. Representative species from fishes (zebrafish, *Danio rerio*; icefish, *Protosalanx chinensis*; Australian ghostshark; *Callorhinchus milii*; sea lamprey, *Petromyzon marinus*), amphibians (western clawed frog, *Xenopus tropicalis*), reptiles (mainland tiger snake, *Notechis scutatus*), birds (chicken, *Gallus gallus*), and mammals (human, *Homo sapiens*) are shown. See also Figures S6 and S7 and Tables S18 and S19 and Data S1.

The gene family history analysis software CAFE (Computational Analysis of gene Family Evolution) cannot identify families created after the most recent common ancestor of the analyzed species (De Bie et al., 2006), that is, gene families that are lineage specific and created in a particular lineage and that may contribute to unique traits (Martin et al., 2010). We identified homologous gene families across 18 fish species using OrthoMCL (Li et al., 2003). When we compared the gene families of 18 fish species (see Figure 1B), we identified 86 single-copy gene families unique to *P. chinensis* (Figure S6; Table S18). Pfam (El-Gebali et al., 2019) and BLAST (Johnson et al., 2008) searches revealed that one of these gene families contain a fibroblast growth factor (FGF) domain (Pfam PF00167) with sequence similarity to *FGF5*. Further manual inspection of the genome assembly and Sanger sequencing of PCR amplicons showed that *P. chinensis* has two distinct *FGF5* gene types (Figure 4A). A three-exon ortholog to *FGF5* to the other fish species was found on scaffold189 (i.e., the canonical *P. chinensis FGF5* gene, denoted *FGF5A*). An *FGF5* gene tree reflected the expected phylogenetic relationship between species (Figure S7). In total, we detected 13 additional copies of *FGF5* in *P. chinensis* (Data S1). Intron sizes of the duplicates ranged from 53 to 6,313 bp. Our data provide an estimate for the number of *FGF5* duplicates in *P. chinensis*; there is a possibility that our analyses did not recover all copies. To determine whether the *P. chinensis FGF5* duplicates are transcribed, we interrogated 14.14Gb RNA sequencing data from a whole animal. Although the number of reads matching *FGF5* was low (2–12 reads), we identified reads corresponding to the exon-intron junction of *FGF5A*, as well as several gene duplicates (Table S19). Therefore, we conclude that *P. chinensis FGF5* duplicates can be transcribed. All *P. chinesis FGF5* genes, including the canonical *P. chinensis FGF5*, would encode C-terminally truncated peptides missing three to eleven of the highly conserved β strands involved in the interaction between *FGF5* and its receptor (see Mohammadi et al., 2005) (Figure 4B). Interestingly, the *P. chinensis FGF5* duplicates are conceptually similar to *FGF5*-short (also known as *FGF5-S*), a mammalian exon 2-deleted isoform that encodes a peptide that prevents *FGF1R* activation by wild-type *FGF5* (Daverio et al., 2017; He et al., 2016; Higgins et al., 2014; Ozawa et al., 1998). *FGF5* is broadly expressed in embryonic, but not adult, tissues of vertebrates. *FGF5* and its receptor play a role in zebrafish development, including neural development during the transition from a larva to an adult (Leerberg et al., 2019; Vemaraju et al., 2012). It is also plausible that *FGF5* is required for scale development, given that there is evidence to suggest that shared development pathways regulate the scales of bony fishes and the hair of mammals. For example, *EDAR* (see above section) regulates hair development in mammals and adult structures such as scales and fins in fish (Aman et al., 2018; Brunsdon and Patton, 2018). Similarly, *FGF5* is a regulator of hair growth in mammals (Daverio et al., 2017; He et al., 2016; Higgins et al., 2014; Ozawa et al., 1998). We speculate that a blunted *FGF5* axis contributes to the retention of larval features by *P. chinensis* but appreciate the need for further studies.

### Contraction of Immune System Genes

Fish larvae have a poorly developed immune system (Vadstein et al., 2013). We found no evidence of positive selection of immune-associated genes, or the skeletal system and other gene ontologies and pathways, in *P. chinensis* (Table S20). In order to gain additional insights into the immune system of *P. chinensis*, we performed gene family gain-and-loss analysis using CAFE (De Bie et al., 2006) and observed four expanded and 69 contracted gene families in *P. chinensis* (Figure 1B; Tables S21 and S22). The contracted gene families include the immune signaling pathways NOD-like receptor signaling (*p* = 9.65×10^−218^), autoimmune thyroid disease (*p* = 5.82×10^−60^), NF-kappa β signaling (*p* = 5.68×10^−48^), and B cell (antigen) receptor signaling (*p* = 3.76×10^−45^). *P. chinensis* and other teleosts have a similar number of genes in most immune system signaling pathways, except for three pathways where *P. chinensis* has a lower number of genes: complement and coagulation cascades (KEGG pathway map04610; 61 genes), antigen processing and presentation (map04612; 61 genes), and intestinal immune network for IgA production (map04672; 28 genes) (Table S23).

**Figure 5:**
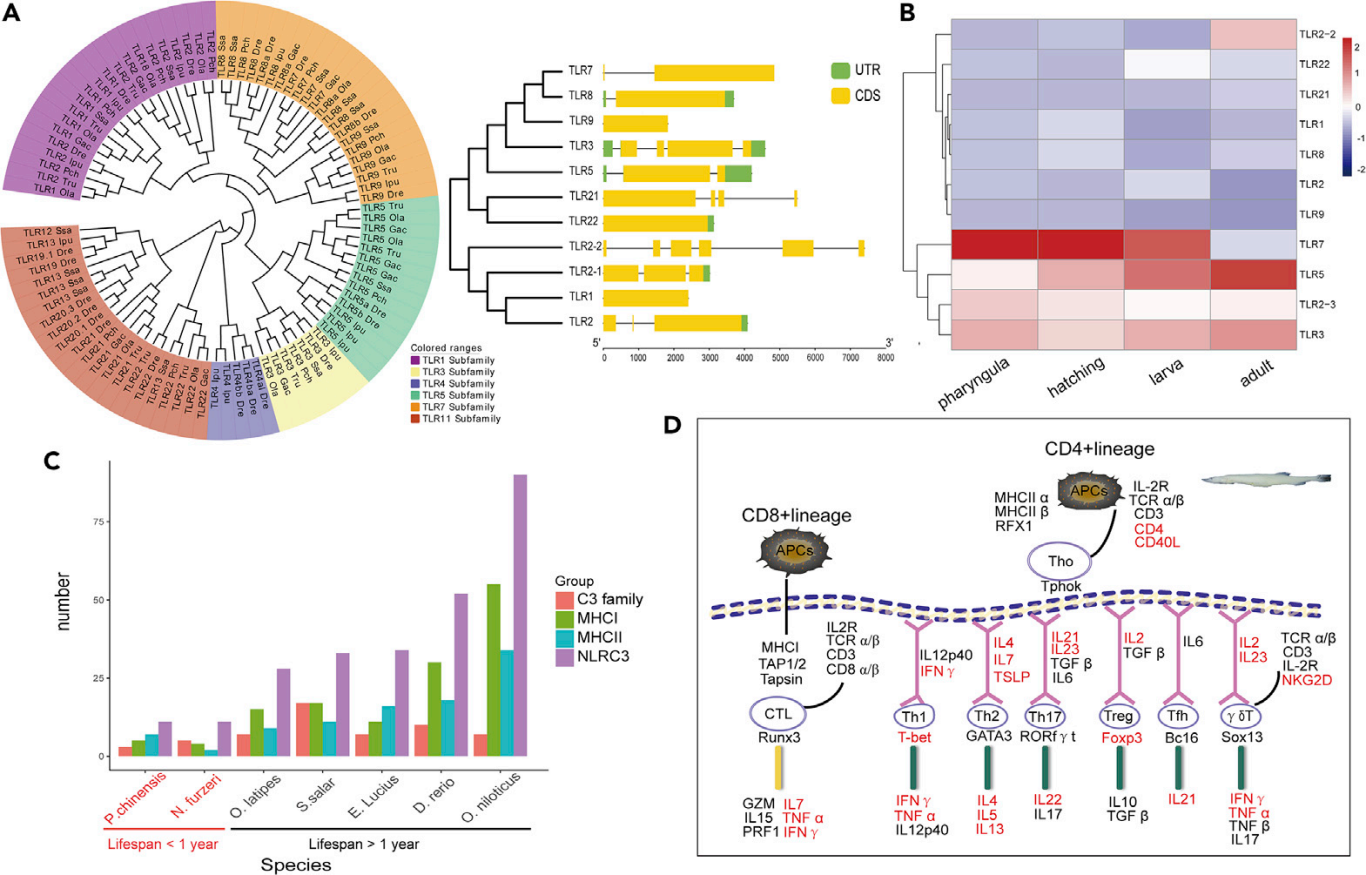
Overview of the *P. chinensis* Immune System Repertoire (A) Left: phylogenetic relationship of the Toll-like receptor (TLR) family genes of *Protosalanx chinensis* (Pch), *Salmo salar* (Sasa), *Danio rerio* (Dre), *Ictalurus punctatus* (Ipu), *Oryzias latipes* (Ola), and *Takifugu rubripes* (Tru). Right: structure of *P. chinensis* TLR genes. Exons are shown as boxes, with coding sequences (CDSs) shown in yellow and untranslated regions (UTRs) in green. (B) Expression in *P. chinensis* of TLR genes at four development stages: pharyngula, hatching, larva, and adult. Gene expression was quantified as reads per kilobase of gene per million mapped reads (RPKM). (C) Overview of the number of genes involved in the complement system C3 family, MHC I protein complex, MHCII protein complex, and NOD-like receptor family (NLRC3) in seven teleost species. (D) Schematic diagram summarizing genes related to different T cell lineages in *P. chinensis*. Genes absent in the genome assembly are indicated in red. See also Figure S8 and Tables S20 and S21–S24.

Toll-like receptors (TLRs) of the innate immune system recognize various pathogen-associated molecular patterns (PAMPs) to activate downstream immune responses (Rebl and Goldammer, 2018). The TLR multigene family comprises a large and variable number (10–15) of genes, and there are substantial sequence differences within and between vertebrate groups, including within teleost fish species (Rebl and Goldammer, 2018; Roach et al., 2005). *P. chinensis* is no exception. For example, *TLR4* is highly divergent in zebrafish and is lost in most teleost species, including, albeit distant, sister taxa to *P. chinensis* (i.e., salmon, trout) (Rebl and Goldammer, 2018; Roach et al., 2005). Based on homology alignment and RefSeq annotations, 11 TLR genes in five sub-families were identified in the *P. chinensis* genome: *TLR1*, *TLR2*, *TLR2-1*, *TLR2-2*, *TLR3*, *TLR5*, *TLR7*, *TLR8*, *TLR9*, *TLR21*, and *TLR22* (Figure 5A). We assessed the expression of *P. chinensis* TLR genes by RNA sequencing from four different development stages (pharyngula, hatching, larva, and adult). *TLR2-3*, *TLR3*, *TLR5*, and *TLR7* were the most highly expressed TLR genes at all stages, suggesting that they play essential roles in the innate immune system of *P. chinensis* (Figure 5B).

The immune response is costly (energy demanding) and comes with life-history trade-offs. Consequently, some small, short-lived animals may have a suppressed or poorly developed immune system and employ terminal investment strategies, i.e., produce as many offspring as possible before an inevitable death (Brace et al., 2017). We counted the genes immune-related families. Short-lived fish species *P. chinensis* (lifespan ∼1 year) and the African turquoise killifish (*Nothobranchius furzeri*; lifespan ∼4 months) have a smaller number of genes in the major histocompatibility complex (MHC) I and II of the adaptive immune system and the NOD-like receptor family of the innate immune system (Figure 5C; Table S24). T cells are at the center of the adaptive immune system. The MHC I machinery allows activation of CD8^+^ T cells upon bacterial infection. IFN-γ (*IFNG*), TNF-α (*TNFA*), and interleukin 7 (*IL-7*) are absent in *P. chinensis* (Figure 5D), whereas *IFNG* and *IL-7* are absent in *N. furzeri* (Figure S8). Genes encoded by T helper cells (T_h_ or CD4^+^) that recognize MHC class II molecules (*IL-2*, *IL-4*, *IL-5*, *IL-13*, *IL-21*, *IL-22*, *IL-23*, *TSLP*, *FOXP3*, and *NKG2D*) are lost in both *P. chinensis* and *N. furzeri* (and *CD40* and *CD40L* are also lost in *P. chinensis*) (Figures 5D and S8). We speculate that the loss of central immune-related genes and lack of associated immunological innovation, as observed in longer-lived teleost (e.g., Malmstrom et al., 2016), reflects the annual life-history strategy of *P. chinensis* and *N. furzeri*. However, given the plasticity of the vertebrate immune system, phylogenetic distances, and the limited number of species examined in this study, larger-scale studies are warranted.

### A Unique Amino Acid Change in the Fish Pigmentation Gene *MPV17* of *P. chinensis*

One of the striking features of Asian icefishes is their transparent body, appearing white postmortem (Roberts, 1984). Loss of pigmentation, a complete loss of pigmentation of either skin and eyes (albinism) or skin alone (leucism), is observed in various fish. These include cave-dwelling species (Borowsky, 2018), as well as lines of zebrafish (D'Agati et al., 2017; Krauss et al., 2013; Tsetskhladze et al., 2012) and medaka (Fukamachi et al., 2001). Melanin-based pigmentation genes are highly conserved in vertebrates (Hubbard et al., 2010), offering an opportunity for comparative genomics analyses. We employed the *P. chinensis* genome assembly and whole-body transcriptome data to examine various genes previously associated with pigmentation loss in fish, including *SLC45A2* (also known as *AIM1* or *MATP*) (Fukamachi et al., 2001; Tsetskhladze et al., 2012), *OCA2* (Gross and Wilkens, 2013; Protas et al., 2006; Yang et al., 2016), *LYST* (Link et al., 2004), and MPV17 (D'Agati et al., 2017; Krauss et al., 2013; Yang et al., 2016). MPV17 (mitochondrial inner membrane protein 17) encodes a mitochondrial channel-forming protein (Calvo et al., 2006; Spinazzola et al., 2006). MPV17 transmembrane domain missense mutations are pathogenic in mammals and cause mitochondrial disorders with which affected individuals die at a young age (El-Hattab et al., 2018; Kim et al., 2016; Lollgen and Weiher, 2015). Mutations of the gene appear to be better tolerated in fish, where they also affect melanin-containing cells (Borowsky, 2018; D'Agati et al., 2017; Martorano et al., 2019). In *P. chinensis* MPV17, we found an amino acid substitution (Q142V) in the terminal fourth transmembrane domain. The glutamine residue is conserved in all other vertebrates examined, from sea lamprey to humans, species with a common ancestor approximately 500 mya (Smith et al., 2013) (Figure 4C). MPV17 transcripts are expressed but have unique changes in transparent zebrafish lines (D'Agati et al., 2017; Krauss et al., 2013) and the cavefish *Sinocyclocheilus anshuiensis* (Yang et al., 2016). A 19-bp deletion of MPV17 coding exons 1 and 2 likely results in pigmentation loss in zebrafish (D'Agati et al., 2017; Krauss et al., 2013). Cavefish in genus *Sinocyclocheilus* have two copies of MPV17, one of which has an in-frame exon deletion and codes for a protein lacking transmembrane four in the albino *S. anshuiensis* (Yang et al., 2016). Similarly, *P. chinensis MPV17* is transcribed (data not shown). The *P. chinensis* MPV17 mutation, a change from a polar glutamine to a non-polar valine, is predicted to affect protein stability by I-Mutant 2.0 (Capriotti et al., 2005) and protein function by PANTHER-PSEP (Tang and Thomas, 2016), PolyPhen2 (Adzhubei et al., 2010), and SIFT (Kumar et al., 2009). Missense residue mutations in transmembrane domains may cause membrane protein disassembly (Ng et al., 2012). Taken together, in particular, given the highly conserved nature of MPV17 Gln142 in vertebrates, we speculate that the unique amino acid change in *P. chinensis* contributes to its ostensibly transparent, pigmentless, skin phenotype by encoding a non-functional or dysfunctional protein in the melanin synthesis pathway.

### Limitations of the Study

There are currently no genome assemblies of other species in the neotenic Asian salmoniform family Salangidae (salangids; Asian icefishes), somewhat limiting the scope of current comparative genomic analyses. The neotenic salamander the Mexican axolotl (*Ambystoma mexicanum*) has been studied in the laboratory for centuries and has amassed a significant body of research (including population genomic studies of wild-type and mutant strains) that can be supported by comparative genome research (Crowner et al., 2019; Nowoshilow et al., 2018; Smith et al., 2019). In contrast, no such studies of Asian icefishes exist. Fortunately, as the number of high-quality fish genomes is increasing, with more than 10,000 species projected (including several species of order Osmeriformes) to be sequenced by 2030 (Fan et al., 2019), the genetic basis of enigmatic but less studied species, such as Asian icefishes, is sure to be realized. Karyotype data are not available for *P. chinensis* or other Asian icefish species, but future efforts will include such data and provide chromosome-level genome assemblies. Finally, although gene loss was called after examining the genome assembly and transcriptome data (assembled from short reads), additional methods, such *de novo* assembly of long-read RNA sequencing reads (e.g., on the PacBio or Nanopore platforms) (de la Rubia et al., 2020), should be performed to further validate our results. Our improved *P. chinensis* genome assembly provides a valuable resource and steppingstone toward this goal. With a new genome assembly in hand, the use of *P. chinensis* as a laboratory animal can proceed in earnest.

### Resource Availability

#### Lead Contact

Further information and requests for resources and reagents should be directed to and will be fulfilled by the Lead Contact, Ming Li (lim@ioz.ac.cn).

#### Materials Availability

This study did not generate new unique reagents.

#### Data and Code Availability

The NCBI BioProject accession number for the *P. chinensis* genome project reported in this paper is PRJNA604876. The accession numbers for *FGF5* gene PCR amplicon sequences are GenBank: MT416578–MT416594. The accession numbers for *Hox* gene clusters gene PCR amplicon sequences are GenBank: MT394613–MT394616.

## Methods

All methods can be found in the accompanying Transparent Methods supplemental file.

## Supplemental Information

Document S1. Transparent Methods, Data S1, Figures S1–S8, and Tables S1–S14, S16, S19, S21–S24

Table S15. Gene Copy Number of Major Gene Families Involved in Bone Formation in Fishes, Related to Figure 2 The gene set was obtained from Venkatesh et al. (2014). Teleosts fish (*Protosalanx chinensis*, *Takifugu rubripes*, *Gasterosteus aculeatus*, *Nothobranchius furzeri*, *Xiphophorus maculatus*, *Oryzias latipes*, *Oreochromis niloticus*, *Hippocampus comes*, *Gadus morhu*, *Salmo salar*, *Esox lucius*, *Ictalurus punctatus*, *Danio rerio*, *Scleropages formosus*, *Anguilla rostrata*, and *Lepisosteus oculatus*, and *Latimeria chalumnae*) and the elephant shark (*Callorhinchus milii*) were examined.

Table S17. Overview of Hox Genes in Fish with a Larval Phenotype, Related to Figure 3 *P. chinensis* denotes the Asian icefish (*Protosalanx chinensis*); *Paedocypris* a Southeast Asian dwarf minnow genus (*P. carbunculus* and *P. micromegethes*). “✓” denotes intact genes; “×,” gene loss; “?,” unknown. The canonical *Hox* gene symbol, *Hox* cluster, as well as the corresponding *P. chinensis* gene model, coordinates, and coding sequence are shown.

Table S18. Single Copy-Gene Families Unique to *P. chinensis*, Related to Figure 4 *P. chinensis* was compared with *Gasterosteus aculeatus*, *Danio rerio*, *Lepisosteus oculatus*, *Gadus morhu*, *Takifugu rubripes*, *Nothobranchius furzeri*, *Esox lucius*, *Oreochromis niloticus*, *Xiphophorus maculatus*, *Oryzias latipes*, *Salmo salar*, *Scleropages formosus*, *Anguilla rostrata*, *Latimeria chalumnae*, *Hippocampus comes*, *Ictalurus punctatus*, and *Callorhinchus milii*.

Table S20. Positively Selected Genes in *P. chinensis* and Their Gene Ontology and KEGG Pathway Enrichment, Related to Figure 5 “Pvalue” refers to the nominal *P* value; “AdjustedPv,” Benjamini-Hochberg-corrected *P* value.
