# Supplementary material for: Insights into the Evolution of Neoteny from the Genome of the Asian Icefish Protosalanx chinensis
Source: iScience. 2020 Jun 14;23(7):101267. doi: 10.1016/j.isci.2020.101267 (PMC7327861; doi:10.1016/j.isci.2020.101267)
Supplement: Document S1. Transparent Methods, Data S1, Figures S1–S8, and Tables S1–S14, S16, S19, S21–S24 [file mmc1.pdf]

iScience, Volume *23*

Supplemental Information

Insights into the Evolution of Neoteny from the Genome of the Asian Icefish *Protosalanx chinensis*

Jie Zhang, Jiwei Qi, Fanglei Shi, Huijuan Pan, Meng Liu, Ran Tian, Yuepan Geng, Huaying Li, Yujie Qu, Jinping Chen, Inge Seim, and Ming Li

SUPPLEMENTAL DATA ITEMS

Supplemental figures

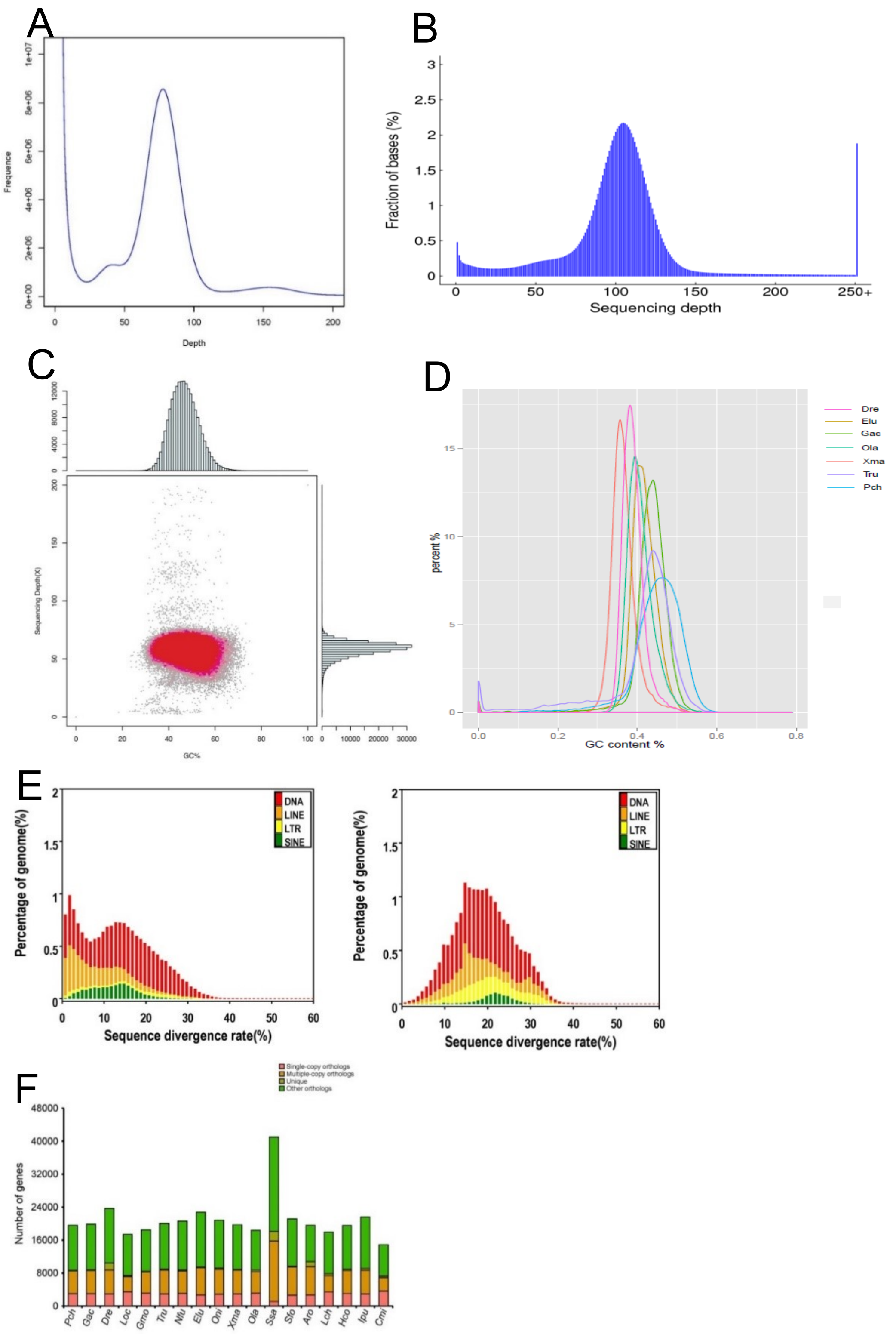

Supplemental Figure S1. ***P. chinensis*** **genome assembly assessment, Related to Figure 1.**

(A) Distribution of 17-mer frequency of filtered Illumina reads mapped to the *P. chinensis* genome. The *y*-axis shows frequency (in millions); the *x*-axis, *k*-mer depth.

(B) Depth distribution of fraction bases. Short-insert reads were mapped to the *P. chinensis* genome assembly using bwa [82]. The *x*-axis shows sequencing depth, the *y*-axis the fraction of bases.

(C) The GC content of the *P. chinensis* genome. The *x*-axis represents GC content; the *y*-axis average sequencing depth. We used a 50 kb non-overlapping sliding window. A lower depth 'island' on the scatter plot is due to sex chromosomes with half the sequencing depth of autosomes.

(D). GC content in the genomes of *P. chinensis* and six other fish species

Pch, *P. chinensis*; Dre, *Danio rerio*; Elu, *Esox lucius*; Gac, *Gasterosteus aculeatus*; Ola, *Oryzias latipes*; Xma, *Xiphophorus maculatus*; Tru, *Takifugu rubric*.

(E) Divergence distribution of transposable elements families in the *P. chinensis* genome. The divergence rate was calculated based on an alignment between RepeatMasker-annotated repeat copies and the consensus sequence in the repeat library.

(F). Orthology delineation among the protein-coding gene family repertoires of *P. chinensis* and 18 other fish species. Pch, *Protosalanx chinensis*; Gac, *Gasterosteus aculeatus*; Dre, *Danio rerio*; Loc, *Lepisosteus oculatus*; Gmo, *Gadus morhu*; Tru, *Takifugu rubripes*; Nfu, *Nothobranc hius furzeri*; Elu, *Esox lucius*; Oni, *Oreochromis niloticus*; Xma, *Xiphophorus maculatus*; Ola, *Oryzias latipes*; Ssa, *Salmo salar*; Sfo, *Scleropages formosus*; Aro, *Anguilla rostrata*; Lch, *Latimeria chalumnae*; Hco, *Hippocampus comes*; Ipu, *Ictalurus punctatus*; Cmi, *Callorhinchus milii*.

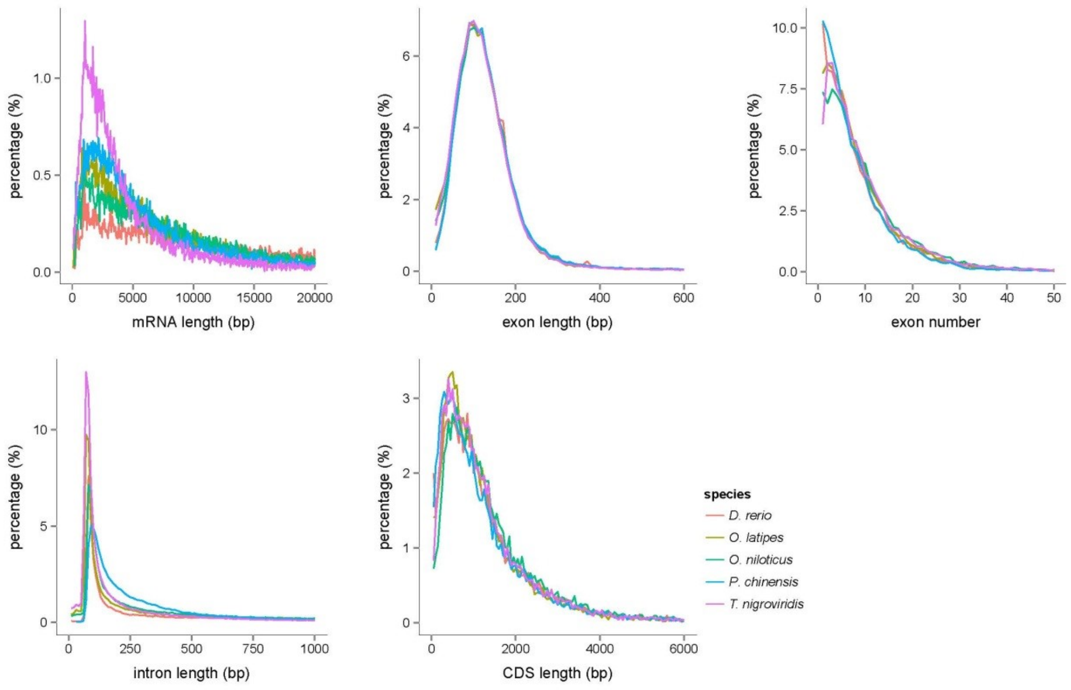

Supplemental Figure S2. A comparison of teleost gene parameters. Characteristic of predicted protein-coding genes in *P. chinensis* genome, Related to Figure 1. Note that mRNA includes untranslated regions (UTRs).

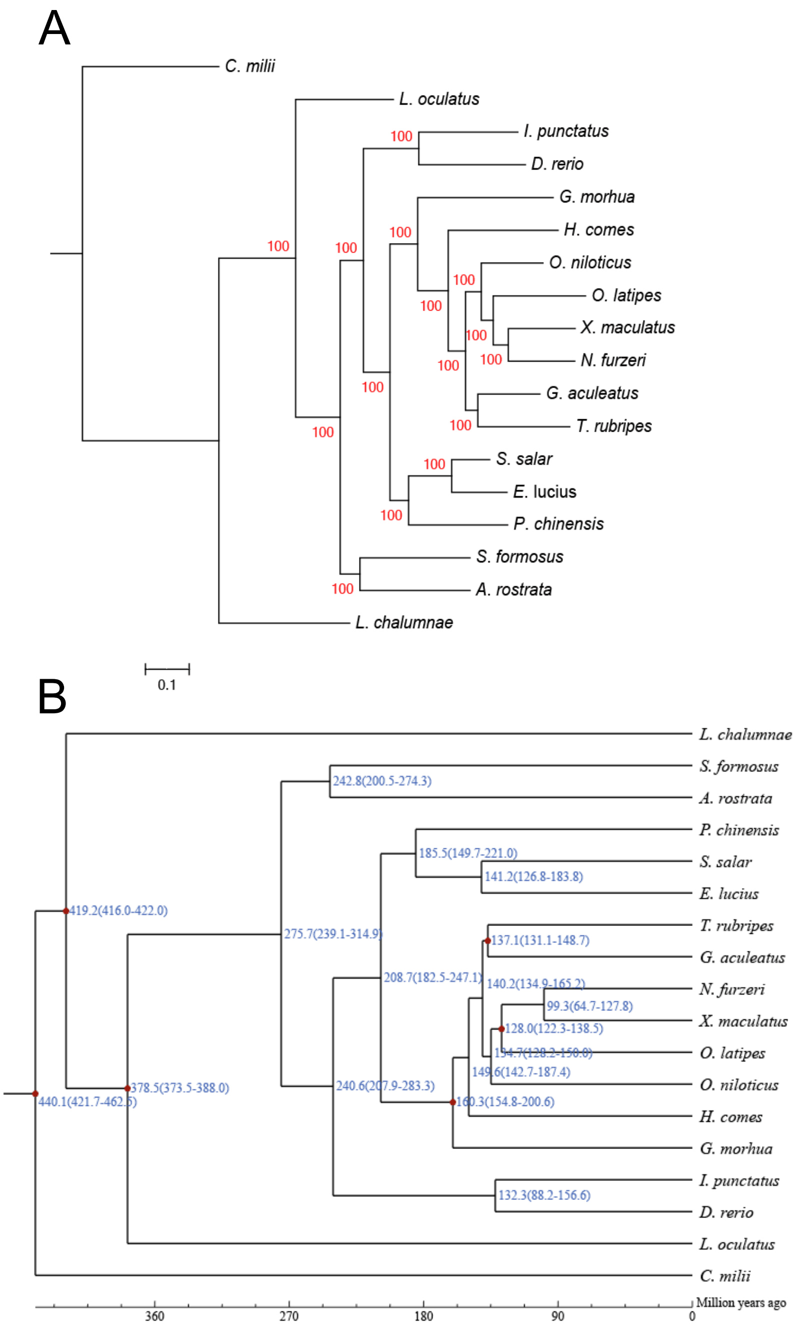

Supplemental Figure S3. Phylogenetic relationship of *P. chinensis* with 17 other fish species, Related to Figure 1.

(A) A maximum likelihood tree generated using RaxML (100 bootstrap replicates) from a 627 single-copy orthologs (coding sequence, CDS) concatenated into a 1,414,350 bp alignment.

(B) Estimated divergence times of 18 fish species. The numbers on nodes represent the divergence times from present (million years ago, Mya).

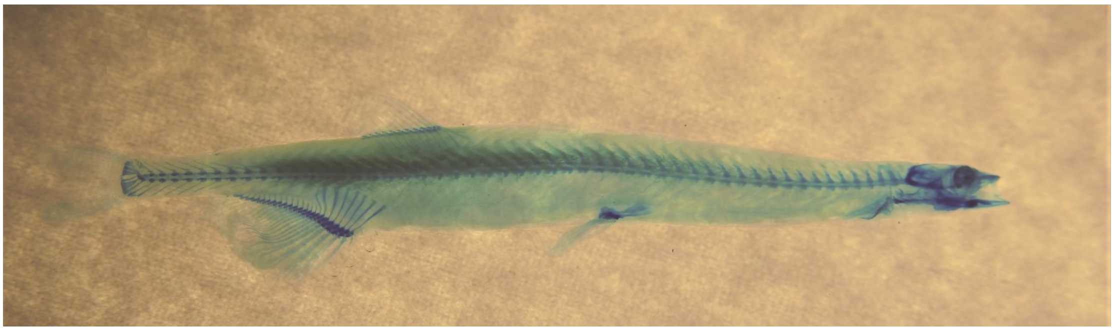

Supplemental Figure S4. ***P. chinensis*** **transparent bone stained specimens, Related to Figure 2.**

After alizarin red and alcian blue staining, endoskeleton of *P. chinensis* is composed of cartilage stained with alcian blue.

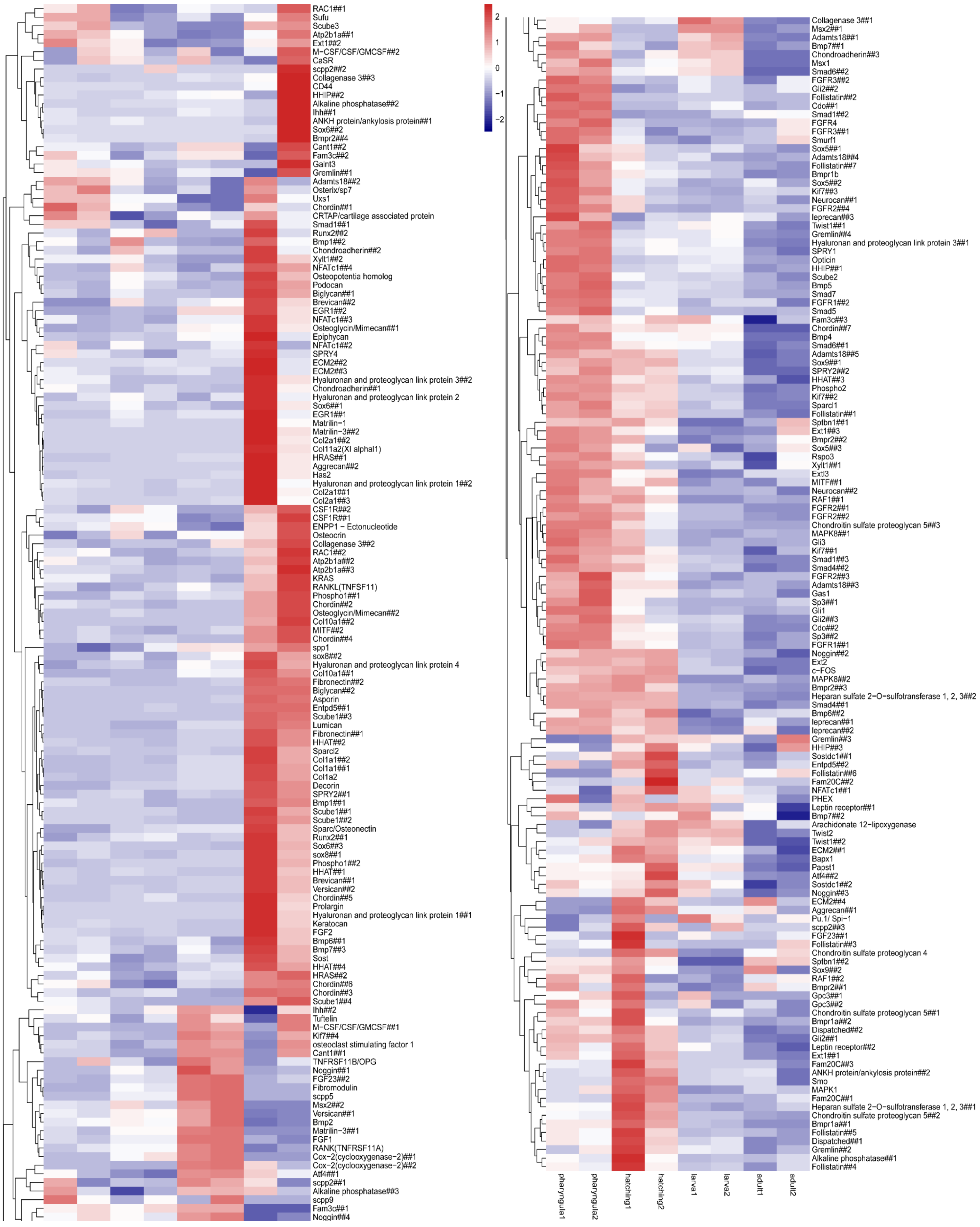
RAC1##1
Sufu
Scube3
Atp2b1a##1
Ext1##2
M−CSF/CSF/GMCSF##2
CaSR
scpp2##2
Collagenase 3##3
CD44
HHIP##2
Alkaline phosphatase##2
Ihh##1
ANKH protein/ankylosis protein##1
Sox6##2
Bmpr2##4
Cant1##2
Fam3c##2
Galnt3
Gremlin##1
Adamts18##2
Osterix/sp7
Uxs1
Chordin##1
CRTAP/cartilage associated protein
Smad1##1
Runx2##2
Bmp1##2
Chondroadherin##2
Xylt1##2
NFATc1##4
Osteopontin homolog
Podocan
Biglycan##1
Brevican##2
EGR1##2
NFATc1##3
Osteoglycin/Mimecan##1
Epiphycan
NFATc1##2
SPRY4
ECM2##2
ECM2##3
Hyaluronan and proteoglycan link protein 3##2
Chondroadherin##1
Hyaluronan and proteoglycan link protein 2
Sox6##1
EGR1##1
Matrilin−1
Matrilin−3##2
Col2a1##2
Col11a2(XI alpha1)
HRAS##1
Aggrecan##2
Has2
Hyaluronan and proteoglycan link protein 1##2
Col2a1##1
Col2a1##3
CSF1R##2
CSF1R##1
ENPP1 − Ectonucleotide
Osteocrin
Collagenase 3##2
RAC1##2
Atp2b1a##2
Atp2b1a##3
KRAS
RANKL(TNFSF11)
Phospho1##1
Chordin##2
Osteoglycin/Mimecan##2
Col10a1##2
MITF##2
Chordin##4
spp1
sox8##2
Hyaluronan and proteoglycan link protein 4
Col10a1##1
Fibronectin##2
Biglycan##2
Asporin
Entpd5##1
Scube1##3
Lumican
Fibronectin##1
HHAT##2
Sparcl2
Col1a1##2
Col1a1##1
Col1a2
Decorin
SPRY2##1
Bmp1##1
Scube1##1
Scube1##2
Sparc/Osteonectin
Runx2##1
Sox6##3
sox8##1
Phospho1##2
HHAT##1
Brevican##1
Versican##2
Chordin##5
Prolargin
Hyaluronan and proteoglycan link protein 1##1
Keratocan
FGF2
Bmp6##1
Bmp7##3
Sost
HHAT##4
HRAS##2
Chordin##6
Chordin##3
Scube1##4
Ihh##2
Tuftelin
M−CSF/CSF/GMCSF##1
Kif7##4
osteoclast stimulating factor 1
Cant1##1
TNFRSF11B/OPG
Noggin##1
FGF23##2
Fibromodulin
scpp5
Msx2##2
Versican##1
Bmp2
Matrilin−3##1
FGF1
RANK(TNFRSF11A)
Cox−2(cyclooxygenase−2)##1
Cox−2(cyclooxygenase−2)##2
Atf4##1
scpp2##1
Alkaline phosphatase##3
scpp9
Fam3c##1
Noggin##4
2
1
0
−1
−2
Collagenase 3##1
Msx2##1
Adamts18##1
Bmp7##1
Chondroadherin##3
Msx1
Smad6##2
FGFR3##2
Gli2##2
Follistatin##2
Cdo##1
Smad1##2
FGFR4
FGFR3##1
Smurf1
Sox5##1
Adamts18##4
Follistatin##7
Bmpr1b
Sox5##2
Kif7##3
Neurocan##1
FGFR2##4
Ieprecan##3
Twist1##1
Gremlin##4
Hyaluronan and proteoglycan link protein 3##1
SPRY1
Opticin
HHIP##1
Scube2
Bmp5
Smad7
FGFR1##2
Smad5
Fam3c##3
Chordin##7
Bmp4
Smad6##1
Adamts18##5
Sox9##1
SPRY2##2
HHAT##3
Phospho2
Kif7##2
Sparcl1
Follistatin##1
Sptbn1##1
Ext1##3
Bmpr2##2
Sox5##3
Rspo3
Xylt1##1
Extl3
MITF##1
Neurocan##2
RAF1##1
FGFR2##1
FGFR2##2
Chondroitin sulfate proteoglycan 5##3
MAPK8##1
Gli3
Kif7##1
Smad1##3
Smad4##2
FGFR2##3
Adamts18##3
Gas1
Sp3##1
Gli1
Gli2##3
Cdo##2
Sp3##2
FGFR1##1
Noggin##2
Ext2
c−FOS
MAPK8##2
Bmpr2##3
Heparan sulfate 2−O−sulfotransferase 1, 2, 3##2
Smad4##1
Bmp6##2
Ieprecan##1
Ieprecan##2
Gremlin##3
HHIP##3
Sostdc1##1
Entpd5##2
Follistatin##6
Fam20C##2
NFATc1##1
PHEX
Leptin receptor##1
Bmp7##2
Arachidonate 12−lipoxygenase
Twist2
Twist1##2
ECM2##1
Bapx1
Papst1
Atf4##2
Sostdc1##2
Noggin##3
ECM2##4
Aggrecan##1
Pu.1/ Spi−1
scpp2##3
FGF23##1
Follistatin##3
Chondroitin sulfate proteoglycan 4
Sptbn1##2
Sox9##2
RAF1##2
Bmpr2##1
Gpc3##1
Gpc3##2
Chondroitin sulfate proteoglycan 5##1
Bmpr1a##2
Dispatched##2
Gli2##1
Leptin receptor##2
Ext1##1
Fam20C##3
ANKH protein/ankylosis protein##2
Smo
MAPK1
Fam20C##1
Heparan sulfate 2−O−sulfotransferase 1, 2, 3##1
Chondroitin sulfate proteoglycan 5##2
Bmpr1a##1
Follistatin##5
Dispatched##1
Gremlin##2
Alkaline phosphatase##1
Follistatin##4
pharyngula1
pharyngula2
hatching1
hatching2
larva1
larva2
adult1
adult2

Supplemental Figure S5. Heat map of the expression of 276 involved in the regulation of bone at four *P. chinensis* development stages, Related to Figure 2.

Expression of 276 genes in regulation of bone at four development stages (pharyngula, hatching, larva, and adult). Numbered suffixes (e.g., pharyngula1 and pharyngula2) indicate biological replicates. Mark "#" after gene symbol (e.g., RAC1##1) indicate numbered gene copy number. Gene expression values (TMM-normalized RPKM) were scaled across all samples for each gene. The gene set was obtained from (Venkatesh *et al.*, 2014). The picture is split into two columns.

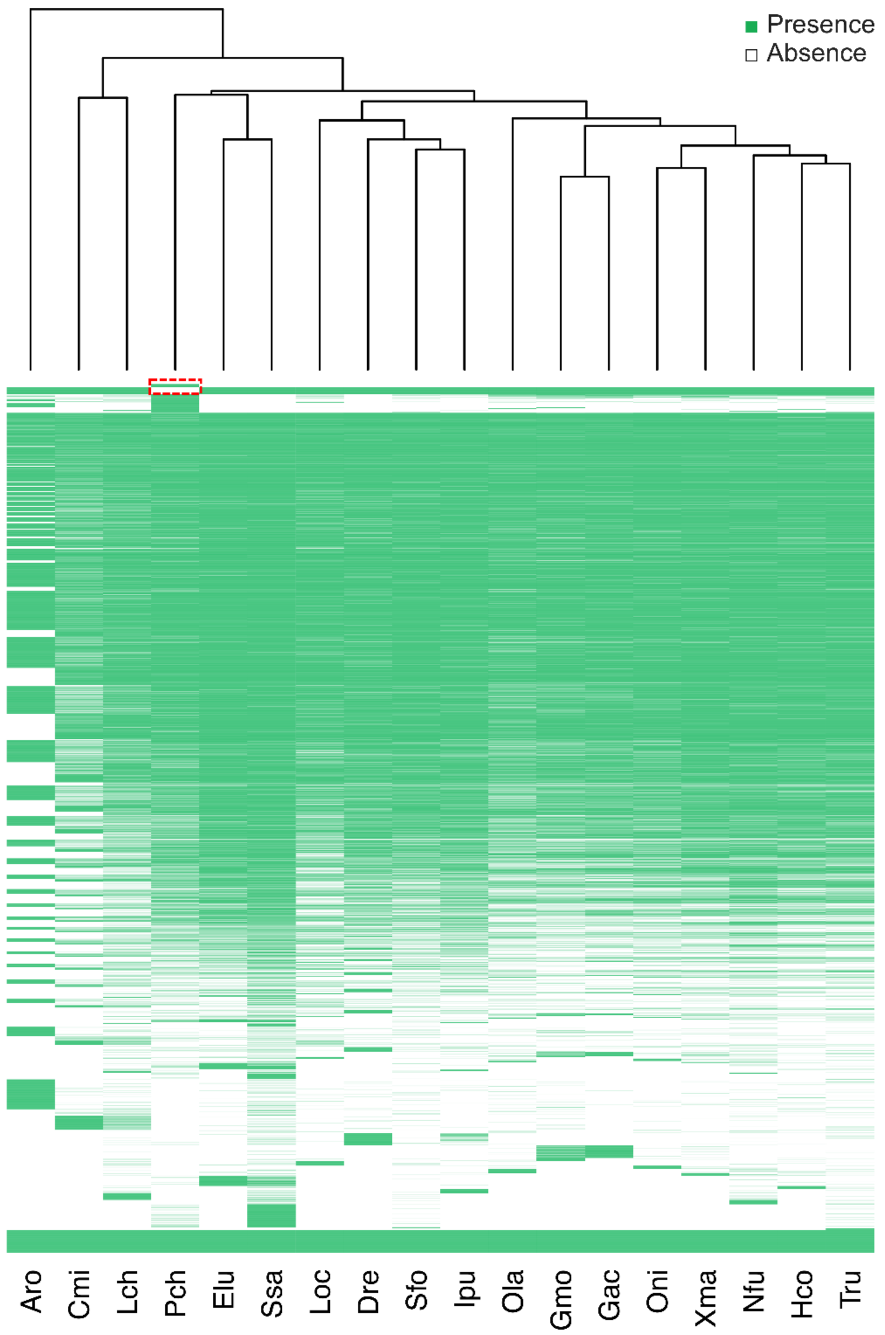

Supplemental Figure S6. Gene family profiles of 18 fish species reveals that 86 single-copy gene families are unique to P. chinensis, Related to Figure 4.

Each column represents a species. Gene family presence is indicated in green; absence in white. Pch, *Protosalanx chinensis*; Gac, *Gasterosteus aculeatus*; Dre, *Danio rerio*; Loc, *Lepisosteus oculatus*; Gmo, *Gadus morhu*; Tru, *Takifugu rubripes*; Nfu, *Nothobranchius furzeri*; Elu, *Esox lucius*; Oni, *Oreochromis niloticus*; Xma, *Xiphophorus maculatus*; Ola, *Oryzias latipes*; Ssa, *Salmo salar*; Sfo,

Scleropages formosus; Aro, *Anguilla rostrata*; Lch, *Latimeria chalumnae*; Hco, *Hippocampus comes*; Ipu, *Ictalurus punctatus*; Cmi, *Callorhinchus milii*. Clustering was performed using Euclidean distance and average linkage parameters.

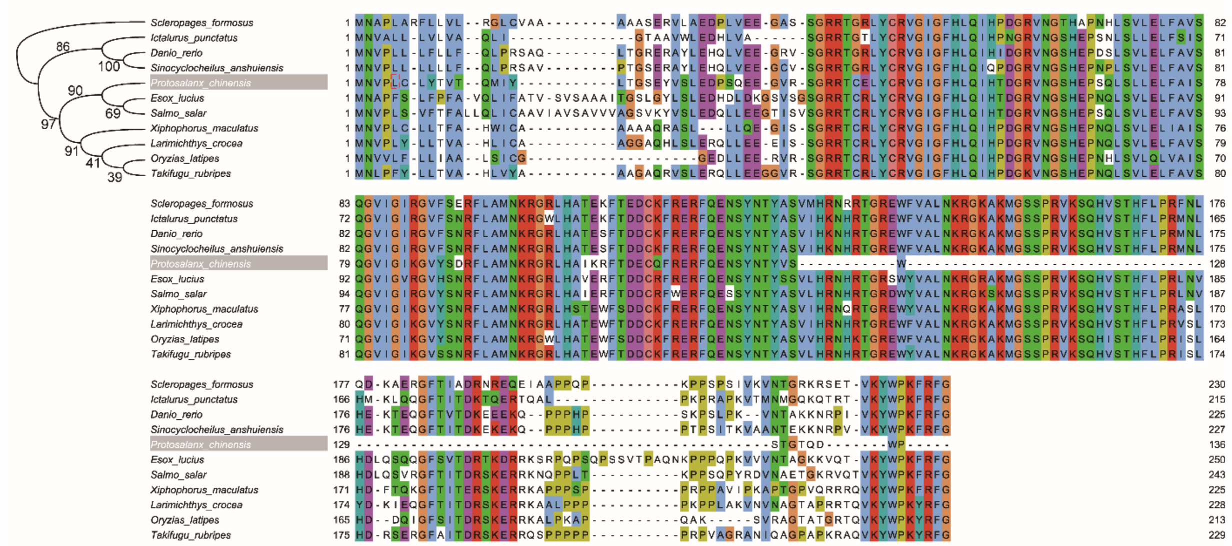

Supplemental Figure S7. ***FGF5 phylogenetic tree and multiple sequence alignment of fish FGF5 proteins*, Related to Figure 4.**

The tree was generated from FGF5 protein sequences. The numbers above branches are ML bootstrap proportion. Protein sequences were aligned using MAFFT.

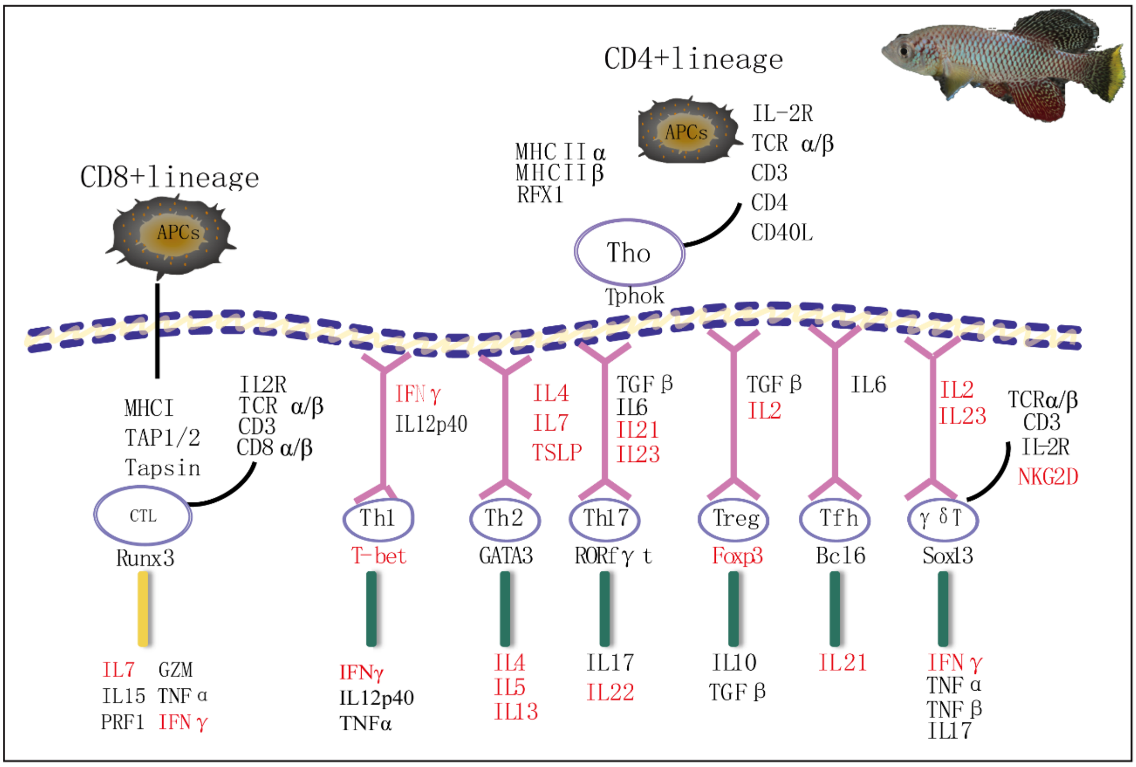

Supplemental Figure S8. Overview of the *N. furzeri* Toll-like receptor family, Related to Figure 5.

Schematic diagram summarizing genes related to different T-cell lineages in *N. furzeri*. Genes absent in the genome assembly are indicated in red.

Supplemental tables

Supplemental Table S1. Summary of genome sequencing strategy, Related to Figure 1.

Paired-end libraries	mate distance	Total data (Gb)	Read length (bp)	Sequence coverage (x)
Illumina	250	26.03	150	53.77
	350	10.67		22.04
	500	14.59		30.14
	2K	32.86		67.88
	5K	12.08		24.95
	10K	17.74		36.64
PacBio	—	10.57	—	21.83
10X Genomics	—	81.92	150	169.22
Total	—	206.46	—	426.48

Supplemental Table S2. Estimated genome size of *P. chinensis* from *k*-mer analysis, Related to Figure 1.

***k*-mer**	**No. *k*-mers**	***k*-mer Depth**	**Genome Size (Mbp)**	**Revised Genome Size (Mbp)**	**Heterozygous Ratio (%)**	**Repeat (%)**
17	38,879,079,724	78	498.45	484.10	0.38	35.43

Supplemental Table S3. ***P. chinensis*** **genome assembly summary statistics, Related to Figure 1.**

	**Length (bp)**		**Number**	
	**Contig (bp)**	**Scaffold(bp)**	**Contig**	**Scaffold**
Total	444,877,745	466,695,321	20,856	1,776
Max	2,137,849	44,188,582	-	-
Number>=2000	-	-	16,493	1,087
N50	103,007	5,188,763	876	23
N60	60,712	4,029,931	1,443	33
N70	34,288	2,574,500	2,425	48
N80	17,547	1,626,991	4,260	71
N90	8,371	794,666	7,943	110

Supplemental Table S4. DNA base composition of the *P. chinensis* genome, Related to Figure 1.

	Number (bp)	% of genome
A	117,329,580	25.14
T	117,357,538	25.14
C	105,042,150	22.51
G	105,148,477	22.53
N	21,817,576	4.67
Total (bp)	466,695,321	100
GC	210,190,627	47.25

Supplemental Table S5. Alignment information of reads mapping to the *P. chinensis* genome, Related to Figure 1.

Reads	Mapping rate	97.89%
Genome	Average sequencing depth	105.77×
	Coverage	92.99%
	Coverage at least 4×	92.27%
	Coverage at least 10×	91.71%
	Coverage at least 20×	91.10%
SNP	Heterozygosis	Percent (%)
	656,664	0.16534
	Homology	Percent (%)
	19,671	0.00164

Supplemental Table S6. Proportion of repeats in the *P. chinensis* genome estimated by various methods, Related to Figure 1.

Type	Repeat size (bp)	% of genome
TRF	60,936,119	13.06
RepeatMasker	123,163,090	26.39
RepeatProteinMask	13,882,973	2.97
Total	149,204,790	31.97

Supplemental Table S7. Statistic of repeat content in the *P. chinensis* genome, Related to Figure 1.

	**Repeatmasker (*de novo* + Repbase)**		**TE Proteins**		**Combined TEs**	
	**Length (bp)**	**%in Genome**	**Length (bp)**	**% in Genome**	**Length (bp)**	**% in Genome**
DNA	48,992,395	10.50	3,856,759	0.83	50,280,383	10.77
LINE	28,924,483	6.20	7,805,974	1.67	30,957,635	6.63
SINE	5,878,163	1.26	0	0.00	5,878,163	1.26
LTR	15,363,100	3.29	2,261,950	0.48	15,787,959	3.38
Other	0	0.00	0	0.00	0	0.00
Satellite	13,959,798	2.99	0	0.00	13,959,798	2.99
Simple repeat	29,638,635	6.35	0	0.00	29,638,635	6.35
Unknown	4,635,326	0.99	0	0.00	4,635,326	0.99
Total	123,163,090	26.39	13,882,973	2.97	125,206,960	26.83

Supplemental Table S8. Assessment of *P. chinensis* genome assembly by mapping of *de novo* assembled transcripts, Related to Figure 1.

Dataset	Number	Total length (bp)	Sequences Covered by assembly (%)	with >90% sequence in one scaffold		with >50% sequence in one scaffold	
				Number	Percent (%)	Number	Percent (%)
>0bp	63,983	44,981,816	98.040	59,229	92.679	62,365	97.471
>200bp	63,983	44,981,816	98.040	59,299	92.679	62,365	97.471
>500bp	26,362	33,379,243	99.560	24,270	92.064	26,083	98.942
>1k	12,418	23,668,832	99.903	11,261	90.683	12,333	99.316
>2k	3,939	11,805,674	99.949	3,464	87.941	3,911	99.289

Supplemental Table S9. CEGMA (Core Eukaryotic Genes Mapping Approach) analysis of *P. chinensis* assemblies, Related to Figure 1.

Species	Reference	complete		complete + partial	
		# Proteins	score (%)	# Proteins	score (%)
Protosalanx chinensis	this study	230	92.74	235	94.76
Protosalanx chinensis	Liu *et al*. (2017)	209	84.27	216	87.10

Supplemental Table S10. BUSCO (Benchmarking Universal Single-Copy Orthologs) analysis of *P. chinensis* assemblies, Related to Figure 1.

Species	Reference	Size (Mbp)	Gene number	BUSCO notation assessment results
Protosalanx chinensis	This study	466.70	23,587	C:93.7%[S:89.6%,D:4.1%],F:2.7%,M:3.6%,n:4584
Protosalanx chinensis	Liu et al. (2017)	536.56	19,884	C:85.6%[S:79.8%,D:5.8%],F:3.5%,M:10.9%,n:4584

Supplemental Table S11. Functional annotation of protein coding genes in the *P. chinensis* genome, Related to Figure 1.

		Number	Percent (%)
**Total**		23,645	-
**Swiss-Prot**		21,757	92.0
**NR**		22,891	96.8
**KEGG**		19,864	84.0
	all	21,299	90.1
**InterPro**	Pfam	18,987	80.3
	GO	15,558	65.8
**Annotated**		22,936	97.0

Supplemental Table S12. Summary of the predicted protein-coding genes in *P. chinensis* genome, Related to Figure 1.

Note that the final gene set includes untranslated (UTR) regions.

Gene set		Number	Average transcript length (bp)	Average CDS length (bp)	Average exons per gene	Average exon length (bp)	Average intron length (bp)
De novo	Augustus	27,786	5,123.06	1,124.17	6.3	178.44	752.31
	Geneid	27,556	11,428.85	1,213.68	6.0	202.28	2,026.79
	Genscan	21,391	15,855.64	1,759.49	9.8	179.54	1,601.94
	GlimmerHMM	84,488	4,703.75	680.05	3.8	178.96	1,418.51
	SNAP	66,823	7,053.04	820.44	5.5	149.17	1,397.18
**Homology**	*Danio rerio*	21,052	7,802.52	1,515.81	8.1	188.09	890.60
	Oryzias latipes	22,166	6,470.85	1,334.08	7.2	186.35	834.00
	Oreochromis niloticus	22,250	7,392.61	1,452.00	7.9	184.56	865.03
	Gasterosteus aculeatus	22,689	6,821.71	1,334.68	7.5	178.78	848.65
	Takifugu rubripes	20,467	7,572.05	1,456.79	8.0	182.77	877.30
	Cynoglossus semilaevis	20,645	8,144.30	1,572.06	8.4	187.66	890.88

	Tetraodon nigroviridis	19,276	7,630.55	1,445.56	8.2	176.21	858.61
	Larimichthys crocea	21,743	7,832.77	1,531.19	8.2	186.01	871.36
	Salmo salar	26,808	6,669.82	1,417.00	7.1	199.53	860.88
**RNASeq**	Cufflinks	41,385	11,009.96	2,757.41	10.1	273.73	909.52
	PASA	26,639	7,428.80	1,365.34	8.4	162.64	819.93
**EVM**		30,832	7,011.59	1,261.33	7.2	175.72	930.75
**PASA**		30,187	7,327.46	1,318.74	7.5	175.63	923.22
**Final set**		23,645	8,529.84	1,509.75	8.7	173.09	909.08

Supplemental Table S13. Summary of predicted RNA genes and their characteristics, Related to Figure 1.

Type		Copy	Average length (bp)	Total length (bp)	% of genome
**miRNA**		1,327	127.20	168,800	0.036169
**tRNA**		1,382	75.64	104,534	0.022399
**rRNA**	rRNA	95	249.18	23,672	0.005072
	18S	29	331.69	9,619	0.002061
	28S	46	265.02	12,191	0.002612
	5.8S	4	148.25	593	0.000127
	5S	16	79.31	1,269	0.000272
**snRNA**	snRNA	520	175.74	91,387	0.019582
	CD-box	176	106.92	18,818	0.004032
	HACA-box	231	240.58	55,574	0.011908
	splicing	98	143.31	14,044	0.003009

Supplemental Table S14. Divergence time between species, Related to Figure 1.

Mya denotes million years ago.

**Taxon A**	**Taxon B**	**Time-range (Mya)**
Gasterosteus aculeatus	*Takifugu rubripes*	97-151
Scleropages formosus	*Lepisosteus oculatus*	374-390
Nothobranchius furzeri	*Oryzias latipes*	128-153
Gadus morhua	*Gasterosteus aculeatus*	139-158
Latimeria chalumnae	*Danio rerio*	416-422
Callorhinchus milii	*Latimeria chalumnae*	422-463

Supplemental Table S16. Expression of genes involved in scale formation in *P. chinensis*, Related to Figure 2.

Transcripts were assessed by interrogating raw RNA-seq reads and a Trinity assembly.

Gene ID	Gene symbol	Name	Transcript detected?
evm.model.scaffold110.338	*EDA*	ectodysplasin A	✓
evm.model.scaffold5.21_evm.model.scaffold5.24	*EDA*	ectodysplasin A	✓
evm.model.scaffold93.206	*EDAR*	ectodysplasin A receptor	✗
evm.model.scaffold22.181	*FGFR1A*	fibroblast growth factor receptor 1a	✓
evm.model.scaffold171.50	*LEF1*	lymphoid enhancer-binding factor 1	✓
evm.model.scaffold34.272	*TCF7*	transcription factor 7 (T-cell specific, HMG-box)	✓
evm.model.scaffold1622.4	*LAMB3*	laminin, beta 3	✓
evm.model.scaffold159.552	*COL7A1*	collagen, type VII, alpha 1	✓

Supplemental Table S19. Summary of BLAST of *FGF5* exon sequences against raw RNA-seq reads from mixed *P. chinensis* tissue, Related to Figure 4.

Sequences were obtained from a multiple sequence alignment (see Supplemental Data 1). Raw RNA-seq reads (150 bp) were queried using a local instance of sequenceserver v1.0.11 [131] and various regions of *P. chinensis* FGF5 genes: the 142 bp 3' region of exon 1, the 142 bp 5' region of exon 2, and their exon intron junction (82 bp of the 3' region of exon 1 and 47 bp of exon 2). Note that *FGF5A* exon 2 is distinct from exon 2 of *FGF5B* to *FGF5N*.

gene	exon 1	exon 2	exon 1-exon 2 junction
FGF5A	4	12	2
FGF5B	0	0	0
FGF5C	0	0	0
FGF5D	12	0	4
FGF5E	8	4	4
FGF5F	2	4	4
FGF5G	12	4	4
FGF5H	0	4	0
FGF5I	0	0	0
FGF5J	0	0	0
FGF5K	0	0	0
FGF5L	0	0	0
FGF5M	0	0	0
FGF5M	0	0	0

Supplemental Table S21. KEGG enrichment of gene families contracted in *P. chinensis*, Related to Figure 5.

MapID	MapTitle	P-value
map04621	NOD-like receptor signaling pathway	9.65E-218
map05133	Pertussis	6.17E-168
map04740	Olfactory transduction	2.30E-107
map05164	Influenza A	2.59E-100
map05322	Systemic lupus erythematosus	4.83E-66
map04640	Hematopoietic cell lineage	2.61E-63
map05320	Autoimmune thyroid disease	5.82E-60
map05416	Viral myocarditis	1.23E-54
map05323	Rheumatoid arthritis	2.45E-49
map04064	NF-kappa B signaling pathway	5.68E-48
map04662	B cell receptor signaling pathway	3.76E-45
map05162	Measles	3.86E-37
map04672	Intestinal immune network for IgA production	1.41E-35
map05310	Asthma	1.58E-35
map05140	Leishmaniasis	2.04E-31
map04145	Phagosome	4.53E-31
map05330	Allograft rejection	1.35E-28
map05150	Staphylococcus aureus infection	2.32E-28
map04650	Natural killer cell mediated cytotoxicity	4.42E-27
map05202	Transcriptional misregulation in cancer	1.11E-22
map05143	African trypanosomiasis	1.60E-22
map05340	Primary immunodeficiency	1.60E-22
map04666	Fc gamma R-mediated phagocytosis	9.62E-21
map05146	Amoebiasis	8.77E-20
map05414	Dilated cardiomyopathy	2.00E-18
map04020	Calcium signaling pathway	8.52E-18
map04664	Fc epsilon RI signaling pathway	1.17E-17
map04072	Phospholipase D signaling pathway	2.75E-12
map05169	Epstein-Barr virus infection	8.48E-12
map05152	Tuberculosis	7.58E-11
map04530	Tight junction	8.15E-06
map04514	Cell adhesion molecules (CAMs)	2.65E-05
map05130	Pathogenic Escherichia coli infection	5.67E-05
map05144	Malaria	9.38E-05
map05332	Graft-versus-host disease	0.0001135
map04940	Type I diabetes mellitus	0.0003764
map05321	Inflammatory bowel disease (IBD)	0.0004392

map04612	Antigen processing and presentation	0.0022873
map04151	PI3K-Akt signaling pathway	0.0022942
map04540	Gap junction	0.0028647
map04360	Axon guidance	0.0138875

Supplemental Table S22. Gene Ontology enrichment of gene families contracted in *P. chinensis*, Related to Figure 5.

GO_ID	GO_Term	GO_Class	P-value	Adjusted P-value
GO:0006915	apoptotic process	BP	0.002995896	0.009705454
GO:0005488	Binding	MF	5.13E-20	1.99E-18
GO:0030246	carbohydrate binding	MF	4.57E-25	2.84E-23
GO:0007155	cell adhesion	BP	1.83E-11	1.68E-10
GO:0007049	cell cycle	BP	0.001330008	0.004809681
GO:0007166	cell surface receptor signaling pathway	BP	7.54E-26	5.87E-24
GO:0034622	cellular macromolecular complex assembly	BP	1.03E-06	5.70E-06
GO:0044430	cytoskeletal part	CC	4.28E-13	5.11E-12
GO:0015074	DNA integration	BP	0.000686831	0.002670054
GO:0048013	ephrin receptor signaling pathway	BP	2.81E-27	2.91E-25
GO:0004930	G-protein coupled receptor activity	MF	1.55E-30	2.40E-28
GO:0007186	G-protein coupled receptor signaling pathway	BP	5.77E-21	2.56E-19
GO:0005525	GTP binding	MF	6.11E-09	4.63E-08
GO:0003924	GTPase activity	MF	0.008251113	0.021746576
GO:0020037	heme binding	MF	0.000103518	0.000487787
GO:0005833	hemoglobin complex	CC	0.00123812	0.00463922
GO:0007156	homophilic cell adhesion	BP	5.24E-18	1.25E-16
GO:0043232	intracellular non-membrane-bounded organelle	CC	0.000520827	0.002131278
GO:0044446	intracellular organelle part	CC	0.001858952	0.006569705
GO:0043167	ion binding	MF	3.56E-13	4.42E-12
GO:0005506	iron ion binding	MF	0.000706633	0.002713122
GO:0046872	metal ion binding	MF	1.26E-16	2.60E-15
GO:0005874	Microtubule	CC	4.22E-07	2.38E-06
GO:0003774	motor activity	MF	8.13E-11	7.23E-10
GO:0016459	myosin complex	CC	5.37E-14	7.95E-13
GO:0003956	NAD(P)+-protein-arginine ADP-ribosyltransferase activity	MF	1.76E-07	1.09E-06
GO:0017111	nucleoside-triphosphatase activity	MF	0.013076943	0.032277217
GO:0000786	Nucleosome	CC	0.007361972	0.019737702
GO:0006334	nucleosome assembly	BP	0.014565938	0.03511633
GO:0004984	olfactory receptor activity	MF	3.14E-135	9.77E-133
GO:0019825	oxygen binding	MF	0.003111349	0.009880125
GO:0015671	oxygen transport	BP	0.00221137	0.007484359
GO:0006471	protein ADP-ribosylation	BP	3.17E-06	1.73E-05
GO:0005515	protein binding	MF	1.61E-11	1.57E-10
GO:0006461	protein complex assembly	BP	0.000371058	0.001580808
GO:0051258	protein polymerization	BP	2.71E-07	1.62E-06

GO:0032550	purine ribonucleoside binding	MF	0.016652344	0.038875768
GO:0035639	purine ribonucleoside triphosphate binding	MF	0.016410404	0.038875768
GO:0032555	purine ribonucleotide binding	MF	0.017907593	0.039780438
GO:0004872	receptor activity	MF	2.77E-15	5.06E-14
GO:0042981	regulation of apoptotic process	BP	0.003208658	0.009880125
GO:0003964	RNA-directed DNA polymerase activity	MF	0.022639537	0.047897251
GO:0007165	signal transduction	BP	0.00609246	0.017069867
GO:0005200	structural constituent of cytoskeleton	MF	1.47E-10	1.20E-09
GO:0001594	trace-amine receptor activity	MF	6.36E-08	4.50E-07
GO:0046914	transition metal ion binding	MF	0.00626301	0.017391036
GO:0004888	transmembrane signaling receptor activity	MF	3.58E-22	1.86E-20

Supplemental Table S23. Number of genes related to KEGG immunity pathways in *P. chinensis* and eight fish species, Related to Figure 5.

Bold denotes lower number of genes in *P. chinensis*

	Immune pathway	***P. chinensis***	***D. rerio***	***I. punctatus***	***T. rubripes***	***T. nigroviridis***	***L. crocea***	***M. zebra***	***O. latipes***	***X. maculatus***
Map04640	Hematopoietic cell lineage	71	71	97	83	70	90	127	96	75
**Map04610**	Complement and coagulation cascades	61	80	114	100	81	90	111	99	88
Map04611	Platelet activation	202	171	176	182	195	204	197	192	189
Map04620	Toll and Imd signaling pathway	66	49	52	46	55	53	56	49	49
Map04624	Toll-like receptor signaling pathway	110	97	106	103	97	122	142	100	102
Map04621	NOD-like receptor signaling pathway	175	157	213	172	165	212	244	177	192
Map04622	RIG-I-like receptor signaling pathway	76	63	71	68	68	86	85	66	65
Map04623	Cytosolic DNA-sensing pathway	53	42	51	40	43	54	66	46	50
Map04650	Natural killer cell mediated cytotoxicity	126	98	132	121	107	131	146	117	132
Map04660	T cell receptor signaling pathway	156	125	131	125	140	149	144	127	125
**Map04612**	Antigen processing and presentation	61	70	94	70	54	72	110	76	73
Map04658	Th1 and Th2 cell differentiation	106	98	101	108	102	114	133	108	99
Map04659	Th17 cell differentiation	137	112	133	123	124	137	159	125	118
Map04657	IL-17 signaling pathway	104	81	102	94	85	114	119	95	95

Map04662	B cell receptor signaling pathway	105	87	101	97	109	108	108	100	94
Map04664	Fc epsilon RI signaling pathway	92	78	72	85	98	91	82	72	75
Map04666	Fc gamma R-mediated phagocytosis	149	119	127	129	150	134	139	126	136
Map04670	Leukocyte transendothelial migration	202	147	182	196	194	232	212	207	207
**Map04672**	Intestinal immune network for IgA production	28	41	62	37	38	43	73	39	33
Map4062	Chemokine signaling pathway	247	199	219	206	219	237	268	215	236
	Total	2327	1985	2336	2185	2194	2473	2721	2232	2233

Supplemental Table S24. Overview of the number of genes in four immunity families in *P. chinensis* and seven fish species, Related to Figure 5.

**Species**	**MHCI**	**MHCII**	**NLRCs**	**C3 family**
P. chinensis	5	7	11	3
E. lucius	11	16	34	7
N. furzeri	4	2	11	5
S. salar	17	11	33	17
D. rerio	30	18	52	10
O. latipes	15	9	28	7
O. niloticus	55	34	90	7

Supplemental Data 1. Duplicated fibroblast growth factor 5 genes in *Protosalanx chinensis*, Related to Figure 4.

(A) Overview of P. *chinensis* FGF5 genes . A neighbor joining tree was generated from conserved sites of a multiple sequence alignment using MAFFT. *FGF5A* denotes the canonical FGF5 gene (exon 1 in brown; exon 2 in green; exon 3 in yellow). Duplicated FGF5 genes (*FGF5B* to *FGFBN*) have a novel exon 2 (shown in blue).

(B) Alignment of proteins encoded by fibroblast growth factor 5 genes in *P. chinensis* and related species in superorder Protacanthopterygii, zebrafish, and human . *Homo sapiens* denotes human; *Danio rerio*, zebrafish, *Salmo salar*, Atlantic salmon; *Esox lucius*, Northern pike. All other sequences are *P. chinensis* genome scaffolds or PCR amplicons. Scaffold189 is the *P. chinensis FGF5A*, the ortholog to teleost *FGF5*, while the sequences below indicate various duplicated *FGF5* genes with a novel exon 2 [see (B)]. The FGF5 domain is indicated by dark blue line underneath the alignment (reside 85 to 219). The location of the 12 -strands are indicted by green boxes. Four residues shared by all FGF genes are indicated in red. Annotations derived from (Mohammadi *et al*., 2005).

(C) Multiple sequence alignment of *P. chinensis FGF5* genes. MAFFT (using the G-INS-i Iterative refinement method) was used to generate multiple sequence alignments of PCR amplicons and genome scaffolds. Exon 1, common to all FGF5 genes, is highlighted in yellow. Alignments from exon 1 onwards exclude *FGF5A*, which employs a different exon 2. The aligned sequence corresponds to the region amplified by PCR of genomic DNA (see transparent Methods). Part of the intron has been omitted.

A

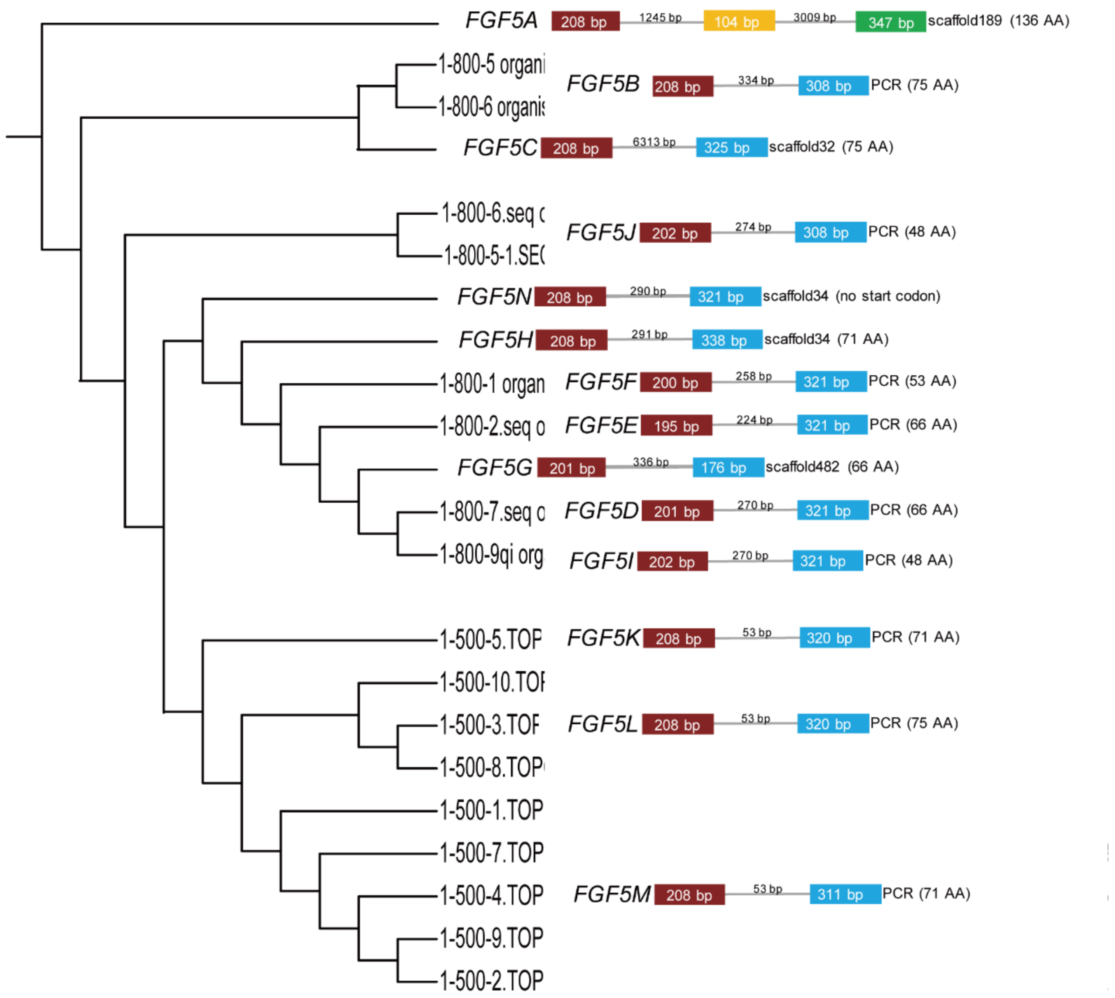
FGF5A 208 bp 1245 bp 104 bp 3009 bp 347 bp scaffold189 (136 AA)
1-800-5 organi
1-800-6 organis
FGF5B 208 bp 334 bp 308 bp PCR (75 AA)
FGF5C 208 bp 6313 bp 325 bp scaffold32 (75 AA)
1-800-6.seq (
1-800-5-1.SE(
FGF5J 202 bp 274 bp 308 bp PCR (48 AA)
FGF5N 208 bp 290 bp 321 bp scaffold34 (no start codon)
FGF5H 208 bp 291 bp 338 bp scaffold34 (71 AA)
1-800-1 organ
FGF5F 200 bp 258 bp 321 bp PCR (53 AA)
1-800-2.seq o
FGF5E 195 bp 224 bp 321 bp PCR (66 AA)
FGF5G 201 bp 336 bp 176 bp scaffold482 (66 AA)
1-800-7.seq o
FGF5D 201 bp 270 bp 321 bp PCR (66 AA)
1-800-9qi org
FGF5I 202 bp 270 bp 321 bp PCR (48 AA)
1-500-5.TOP
FGF5K 208 bp 53 bp 320 bp PCR (71 AA)
1-500-10.TOI
1-500-3.TOP
FGF5L 208 bp 53 bp 320 bp PCR (75 AA)
1-500-8.TOP
1-500-1.TOP
1-500-7.TOP
1-500-4.TOP
FGF5M 208 bp 53 bp 311 bp PCR (71 AA)
1-500-9.TOP
1-500-2.TOP

B

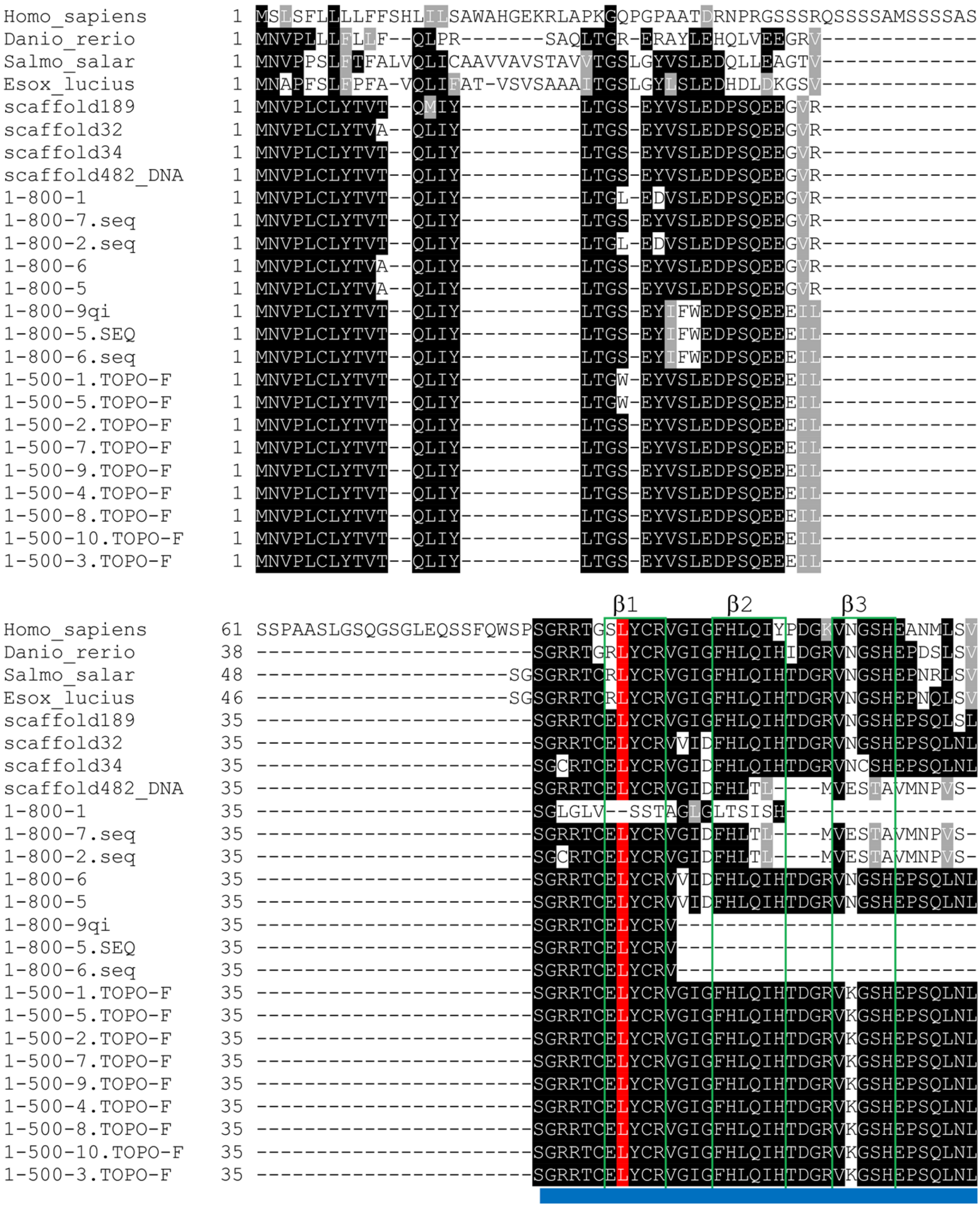
Homo_sapiens 1 MSLSFLLLLFFSHLILSAWAHGEKRLAPKGQPGPAATDRNPRGSSSRQSSSSAMSSSSAS
Danio_rerio 1 MNVPLLLFLLF--QLPR-------SAQLTGR-ERAYLEHQLVEEGRV-------------
Salmo_salar 1 MNVPPSLFTFALVQLICAAVVAVSTAVVTGSLGYVSLEDQLLEAGTV-------------
Esox_lucius 1 MNAPFSLFPFA-VQLIFAT-VSVSAAAITGSLGYISLEDHDLDKGSV-------------
scaffold189 1 MNVPLCLYTVT--QMIY-----------LTGS-EYVSLEDPSQEEGVR------------
scaffold32 1 MNVPLCLYTVA--QLIY-----------LTGS-EYVSLEDPSQEEGVR------------
scaffold34 1 MNVPLCLYTVT--QLIY-----------LTGS-EYVSLEDPSQEEGVR------------
scaffold482_DNA 1 MNVPLCLYTVT--QLIY-----------LTGS-EYVSLEDPSQEEGVR------------
1-800-1 1 MNVPLCLYTVT--QLIY-----------LTGL-EDVSLEDPSQEEGVR------------
1-800-7.seq 1 MNVPLCLYTVT--QLIY-----------LTGS-EYVSLEDPSQEEGVR------------
1-800-2.seq 1 MNVPLCLYTVT--QLIY-----------LTGL-EDVSLEDPSQEEGVR------------
1-800-6 1 MNVPLCLYTVA--QLIY-----------LTGS-EYVSLEDPSQEEGVR------------
1-800-5 1 MNVPLCLYTVA--QLIY-----------LTGS-EYVSLEDPSQEEGVR------------
1-800-9qi 1 MNVPLCLYTVT--QLIY-----------LTGS-EYIFWEDPSQEEEIL------------
1-800-5.SEQ 1 MNVPLCLYTVT--QLIY-----------LTGS-EYIFWEDPSQEEEIL------------
1-800-6.seq 1 MNVPLCLYTVT--QLIY-----------LTGS-EYIFWEDPSQEEEIL------------
1-500-1.TOPO-F 1 MNVPLCLYTVT--QLIY-----------LTGW-EYVSLEDPSQEEEIL------------
1-500-5.TOPO-F 1 MNVPLCLYTVT--QLIY-----------LTGW-EYVSLEDPSQEEEIL------------
1-500-2.TOPO-F 1 MNVPLCLYTVT--QLIY-----------LTGS-EYVSLEDPSQEEEIL------------
1-500-7.TOPO-F 1 MNVPLCLYTVT--QLIY-----------LTGS-EYVSLEDPSQEEEIL------------
1-500-9.TOPO-F 1 MNVPLCLYTVT--QLIY-----------LTGS-EYVSLEDPSQEEEIL------------
1-500-4.TOPO-F 1 MNVPLCLYTVT--QLIY-----------LTGS-EYVSLEDPSQEEEIL------------
1-500-8.TOPO-F 1 MNVPLCLYTVT--QLIY-----------LTGS-EYVSLEDPSQEEEIL------------
1-500-10.TOPO-F 1 MNVPLCLYTVT--QLIY-----------LTGS-EYVSLEDPSQEEEIL------------
1-500-3.TOPO-F 1 MNVPLCLYTVT--QLIY-----------LTGS-EYVSLEDPSQEEEIL------------
β1
β2
β3
Homo_sapiens 61 SSPAASLGSQGSGLEQSSFQWSPSGRRTGSLYCRVGIGFHLQIYPDGKVNGSHEANMLSV
Danio_rerio 38 -----------------------SGRRTGRLYCRVGIGFHLQIHIDGRVNGSHEPDSLSV
Salmo_salar 48 ---------------------SGSGRRTCRLYCRVGIGFHLQIHTDGRVNGSHEPNRLSV
Esox_lucius 46 ---------------------SGSGRRTCRLYCRVGIGFHLQIHTDGRVNGSHEPNQLSV
scaffold189 35 -----------------------SGRRTCELYCRVGIGFHLQIHTDGRVNGSHEPSQLSL
scaffold32 35 -----------------------SGRRTCELYCRVVIDFHLQIHTDGRVNGSHEPSQLNL
scaffold34 35 -----------------------SGCRTCELYCRVGIDFHLQIHTDGRVNCSHEPSQLNL
scaffold482_DNA 35 -----------------------SGRRTCELYCRVGIDFHLTL----MVESTAVMNPVS-
1-800-1 35 -----------------------SGLGLV--SSTAGLGLTSISH----------------
1-800-7.seq 35 -----------------------SGRRTCELYCRVGIDFHLTL----MVESTAVMNPVS-
1-800-2.seq 35 -----------------------SGCRTCELYCRVGIDFHLTL----MVESTAVMNPVS-
1-800-6 35 -----------------------SGRRTCELYCRVVIDFHLQIHTDGRVNGSHEPSQLNL
1-800-5 35 -----------------------SGRRTCELYCRVVIDFHLQIHTDGRVNGSHEPSQLNL
1-800-9qi 35 -----------------------SGRRTCELYCRV-------------------------
1-800-5.SEQ 35 -----------------------SGRRTCELYCRV-------------------------
1-800-6.seq 35 -----------------------SGRRTCELYCRV-------------------------
1-500-1.TOPO-F 35 -----------------------SGRRTCELYCRVGIGFHLQIHTDGRVKGSHEPSQLNL
1-500-5.TOPO-F 35 -----------------------SGRRTCELYCRVGIGFHLQIHTDGRVKGSHEPSQLNL
1-500-2.TOPO-F 35 -----------------------SGRRTCELYCRVGIGFHLQIHTDGRVKGSHEPSQLNL
1-500-7.TOPO-F 35 -----------------------SGRRTCELYCRVGIGFHLQIHTDGRVKGSHEPSQLNL
1-500-9.TOPO-F 35 -----------------------SGRRTCELYCRVGIGFHLQIHTDGRVKGSHEPSQLNL
1-500-4.TOPO-F 35 -----------------------SGRRTCELYCRVGIGFHLQIHTDGRVKGSHEPSQLNL
1-500-8.TOPO-F 35 -----------------------SGRRTCELYCRVGIGFHLQIHTDGRVKGSHEPSQLNL
1-500-10.TOPO-F 35 -----------------------SGRRTCELYCRVGIGFHLQIHTDGRVKGSHEPSQLNL
1-500-3.TOPO-F 35 -----------------------SGRRTCELYCRVGIGFHLQIHTDGRVKGSHEPSQLNL

<CONTINUED>

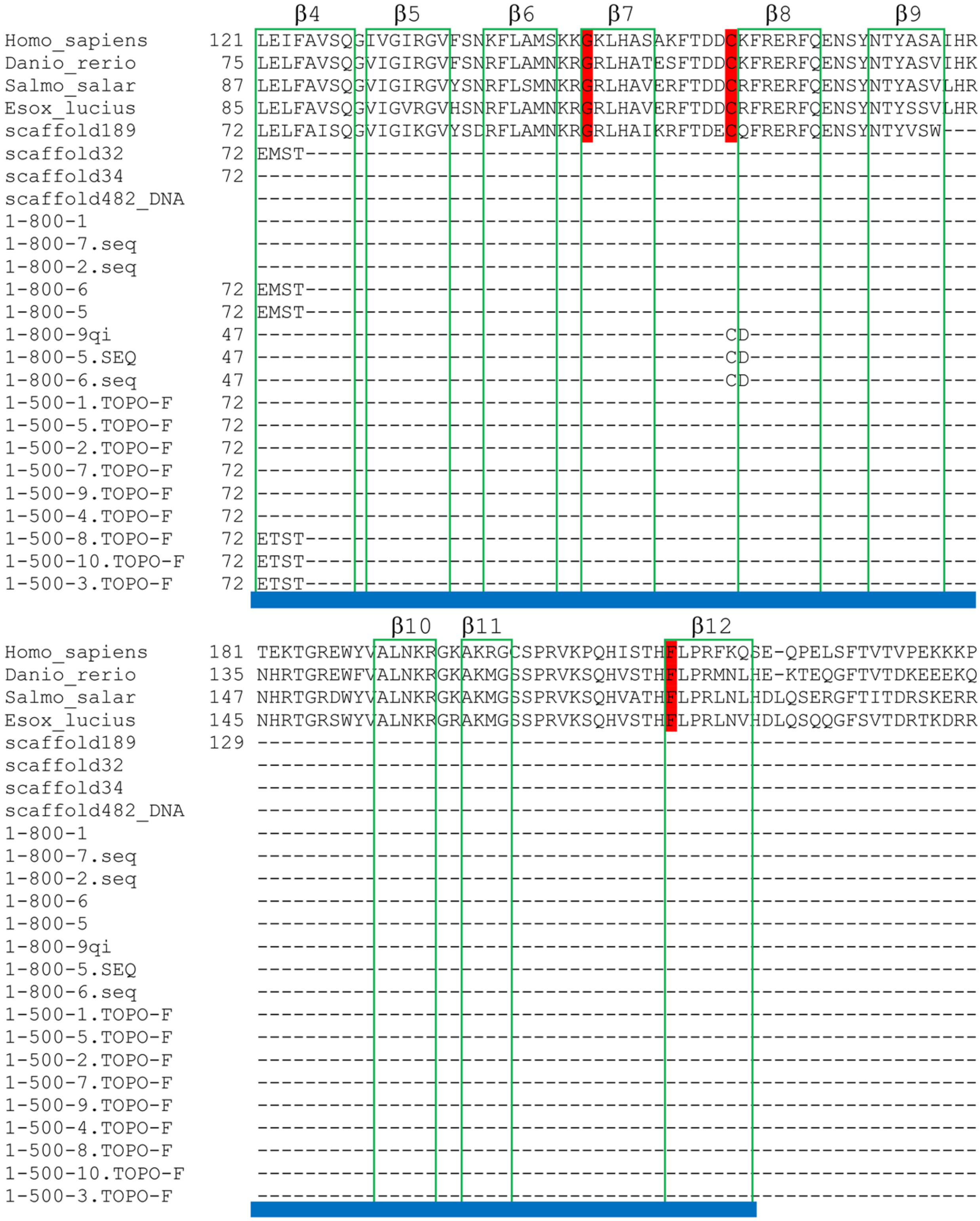

β4 β5 β6 β7 β8 β9
Homo_sapiens 121 LEIFAVSQGIVGIRGVFSNKFLAMSKKGKLHASAKFTDDCKFRERFQENSYNTYASAIHR
Danio_rerio 75 LELFAVSQGVIGIRGVFSNRFLAMNKRGRLHATESFTDDCKFRERFQENSYNTYASVIHK
Salmo_salar 87 LELFAVSQGVIGIRGVYSNRFLSMNKRGRLHAVERFTDDCRFRERFQENSYNTYASVLHR
Esox_lucius 85 LELFAVSQGVIGVRGVHSNRFLAMNKRGRLHAVERFTDDCRFRERFQENSYNTYSSVLHR
scaffold189 72 LELFAISQGVIGIKGVYSDRFLAMNKRGRLHAIKRFTDECQFRERFQENSYNTYVSW---
scaffold32 72 EMST--
scaffold34 72 --
scaffold482_DNA --
1-800-1 --
1-800-7.seq --
1-800-2.seq --
1-800-6 72 EMST--
1-800-5 72 EMST--
1-800-9qi 47 ---------------------------------------CD-------------------
1-800-5.SEQ 47 ---------------------------------------CD-------------------
1-800-6.seq 47 ---------------------------------------CD-------------------
1-500-1.TOPO-F 72 --
1-500-5.TOPO-F 72 --
1-500-2.TOPO-F 72 --
1-500-7.TOPO-F 72 --
1-500-9.TOPO-F 72 --
1-500-4.TOPO-F 72 --
1-500-8.TOPO-F 72 ETST--
1-500-10.TOPO-F 72 ETST--
1-500-3.TOPO-F 72 ETST--
β10 β11 β12
Homo_sapiens 181 TEKTGREWYVALNKRGKAKRGCSPRVKPQHISTHFLPRFKQSE-QPELSFTVTVPEKKKP
Danio_rerio 135 NHRTGREWFVALNKRGKAKMGSSPRVKSQHVSTHFLPRMNLHE-KTEQGFTVTDKEEEKQ
Salmo_salar 147 NHRTGRDWYVALNKRGKAKMGSSPRVKSQHVATHFLPRLNLHDLQSERGFTITDRSKERR
Esox_lucius 145 NHRTGRSWYVALNKRGRAKMGSSPRVKSQHVSTHFLPRLNVHDLQSQQGFSVTDRTKDRR
scaffold189 129 --
scaffold32 --
scaffold34 --
scaffold482_DNA --
1-800-1 --
1-800-7.seq --
1-800-2.seq --
1-800-6 --
1-800-5 --
1-800-9qi --
1-800-5.SEQ --
1-800-6.seq --
1-500-1.TOPO-F --
1-500-5.TOPO-F --
1-500-2.TOPO-F --
1-500-7.TOPO-F --
1-500-9.TOPO-F --
1-500-4.TOPO-F --
1-500-8.TOPO-F --
1-500-10.TOPO-F --
1-500-3.TOPO-F --

C

[EXON 1]

```
M gttcctctttgtctttataccgtcacccagttgatttacctgactggatgggagtatgtt
M gttcctctttgtctttataccgtcacccagttgatttacctgactggatcggagtatgtt
M gttcctctttgtctttataccgtcacccagttgatttacctgactggatcggagtatgtt
M gttcctctttgtctttataccgtcacccagttgatttacctgactggatcggagtatgtt
M gttcctctttgtctttataccgtcacccagttgatttacctgactggatcggagtatgtt
L gttcctctttgtctttataccgtcactcagttgatttacctgactggatcggagtatgtt
L gttcctctttgtctttataccgtcactcagttgatttacctgactggatcggagtatgtt
L gttcctctttgtctttataccgtcactcagttgatttacctgactggatcggagtatgtt
K gttcctctttgtctttataccgtcactcagttgatttacctgactggatgggagtatgtt
I gttcctctttgtctttataccgtcactcagttgatttacctgactggatcggagtatatt
D gttcctctttgtctttataccgtcactcagttgatttacctgactggatcggagtatgtt
E gttcctctttgtctttataccgtcactcagttgatttacctgactggattggaggatgtt
F gttcctctttgtctttataccgtcactcagttgatttacctgactggattggaggatgtt
H gttcctctttgtctttataccgtcactcagttgatttacctgactggatcggagtatgtt
B gttcctctttgtctttataccgtcgctcagttgatttacctgactggatcggagtatgtt
B gttcctctttgtctttataccgtcgctcagttgatttacctgactggatcggagtatgtt
J gttcctctttgtctttataccgtcactcagttgatttacctgactggatcggagtatatt
J gttcctctttgtctttataccgtcactcagttgatttacctgactggatcggagtatatt
G gttcctctttgtctttataccgtcacccagttgatttacctgactggatcggagtatgtt
N ctcctgtgtgggcccgttaccgaagacgccgtgccccaaggagcctgttgccttgacttc
C gttcctctttgtctttataccgtcgctcagttgatttacctgactggatcggagtatgtt
A gttcctctttgtctttataccgtcactcagatgatttacctgactggatccgagtacgtt
  *.*. . * * *..  ***** . .    ** ...*  ..*. * *     *. *.

M tctttggaagacccttctcaggaagaggagatcctctcaggacgcaggacttgtgagctc
M tctttggaagacccttctcaggaagaggagatcctctcaggacgcaggacttgtgagctc
M tctttggaagacccttctcaggaagaggagatcctctcaggacgcaggacttgtgagctc
M tctttggaagacccttctcaggaagaggagatcctctcaggacgcaggacttgtgagctc
M tctttggaagacccttctcaggaagaggagatcctctcaggacgcaggacttgtgagctc
L tctttggaagacccttctcaggaagaggagatcctctcaggacgcaggacttgtgagctc
L tctttggaagacccttctcaggaagaggagatcctctcaggacgcaggacttgtgagctc
L tctttggaagacccttctcaggaagaggagatcctctcaggacgcaggacttgtgagctc
K tctttggaagacccttctcaggaagaggaaatcctctcaggacgcaggacttgtgagctc
I ttttgggaagacccttctcaggaggaggagatcctctcaggacgcaggacttgtgagctc
D tctttggaagacccttctcaggaggaggggtccgctcaggacgcaggacttgtgagctc
E tctttggaagacccttctcaggaggaggggtccgctcaggatgcaggacttgtgagctc
F tctttggaagacccttctcaggaggaggggtccgctcagggt-taggacttgtgagctc
H tctttggaagacccttctcaggaggaggggtccgctcaggatgcaggacttgtgagctc
B tctttggaagacccttctcaggaggaggggtgcgctcaggacgcaggacttgtgagctc
B tctttggaagacccttctcaggaggaggggtgcgctcaggacgcaggacttgtgagctc
```

```
J ttttgggaagacccttctcaggaggaggagatcctctcaggacgcaggacttgtgagctc
J ttttgggaagacccttctcaggaggaggagatcctctcaggacgcaggacttgtgagctc
G tctttggaagacccttctcaggaggagggggtccgctcaggacgcaggacttgtgagctc
N tcacctgatgaaggggttagggataaccctagccctaaccctaaccctaaccctagccct
C tctttggaagacccttctcaggaggagggggtgcgctcaggacgcaggacttgtgagctc
A tctttggaagacccttctcaggaggagggggtccgctcaggacgcaggacttgtgagctc
  *. .  ** **    .* .*** .*   .  * .       .  * .. *.. *..

M tactgcaggg-ttgggattggcttccatcttcagattcacaccgatggtagagtcaaagg
M tactgcaggg-ttgggattggcttccatcttcagattcacaccgatggtagagtcaaagg
M tactgcaggg-ttgggattggcttccatcttcagattcacaccgatggtagagtcaaagg
M tactgcaggg-ttgggattggcttccatcttcagattcacaccgatggtagagtcaaagg
M tactgcaggg-ttgggattggcttccatcttcagattcacaccgatggtagagtcaaagg
L tactgcaggg-ttgggattggcttccatcttcagattcacaccgatggtagagtcaaagg
L tactgcaggg-ttgggattggcttccatcttcagattcacaccgatggtagagtcaaagg
L tactgcaggg-ttgggattggcttccatcttcagattcacaccgatggtagagtcaaagg
K tactgcaggg-ttgggattggcttccatcttcagattcacaccgatggtagagtcaaagg
I tactgcagggtttgtgattgacttccatc-------tcacactgatggtagagtcaacgg
D tactgcaggg-ttgggattgacttccatc-------tcacactgatggtagagtcaacgg
E tactgcaggg-ttgggattgacttccatc-------tcacactgatggtagagtcaacgg
F tactgcaggg-ttgggattgacttccatc-------tcacactgatggtagagtcaacgg
H tactgcaggg-ttgggattgacttccatcttcagattcacaccgatggtagagtcaactg
B tactgcaggg-ttgtgattgacttccatcttcagattcacaccgatggtagagtcaacgg
B tactgcaggg-ttgtgattgacttccatcttcagattcacaccgatggtagagtcaacgg
J tactgcagggtttgtgattgacttccatc-------tcacaccgatggtagagtcaacgg
J tactgcagggtttgtgattgacttccatc-------tcacaccgatggtagagtcaacgg
G tactgcaggg-ttgggattgacttccatc-------tcacactgatggtagagtcaacgg
N aaccctaacc-atggagaggatcaccatgatcagattcacaccgatggtagagtcaacgg
C tactgcaggg-ttgtgattgacttccatcttcagattcacaccgatggtagagtcaacgg
A tactgcagag-ttgggattggcttccatcttcagattcacaccgatggtagagtcaacgg
   **. .*.   ** ..  *... ****       ******.**************  *

M cagtcatgaacccagtcagttaagtg----------------------------------
M cagtcatgaacccagtcagttaagtg----------------------------------
M cagtcatgaacccagtcagttaagtg----------------------------------
M cagtcatgaacccagtcagttaagtg----------------------------------
M cagtcatgaacccagtcagttaagtg----------------------------------
L cagtcatgaacccagtcagttaagtg----------------------------------
L cagtcatgaacccagtcagttaagtg----------------------------------
L cagtcatgaacccagtcagttaagtg----------------------------------
K cagtcatgaacccagtcagttaagtg----------------------------------
I cagtcatgaatccagtcagttaagtaagtttcattcatttattacatgcatccaacaggt
D cagtcatgaatccagtcagttaagtaagtttcattcatttattacatgcatccaacaggt
E cagtcatgaatccagtcagttaagtaaatttcattcatttattacatgcatccaacaggt
```

```
F cagtcatgaatccagtcagttaagtaagtttcattcatttattacatgcatccaacaggt
H cagtcatgaacccagtcagttaagtaagtttcattcatttattacatgcatccaacaggt
B cagtcatgaacccagtcagttaagtacgtttcattcattcattacatgcatccaacaggt
B cagtcatgaacccagtcagttaagtacgtttcattcattcattacatgcatccaacaggt
J cagtcatgaacccagtcagttaagtaagtttcattcattcattacatgcatccaacaggt
J cagtcatgaacccagtcagttaagtaagtttcattcattcattacatgcatccaacaggt
G cagtcatgaatccagtcagttaagtaagtttcattcatttattacatgcatccaacaggt
N cagtcatgaacccagtcagttaagtaagtttcattcattcattacatgcatccaacaggt
C cagtcatgaacccagtcagttaagtacgtttcattcattcattacatgcatccaacaggt
A cagtcatgaacccagtcagttaagtaagtttcatttattcattacatgcatccaacaggt
  **********.**************.

M ------------------------------------------------------------
M ------------------------------------------------------------
M ------------------------------------------------------------
M ------------------------------------------------------------
M ------------------------------------------------------------
L ------------------------------------------------------------
L ------------------------------------------------------------
L ------------------------------------------------------------
K ------------------------------------------------------------
I tattgaaccaagccactcctctccactcctcccctccactcct-----------------
D tattgaaccaagccactcctctccactcctcccctccactcct-----------------
E tattgaaccaagccactcctctgcactcctcccctccactcct-----------------
F tattgaaccaagccactcctctccactcctcccctcctctcct----------tctctcc
H tattgaactccaccccactcctcccctcctctcctccactcctccccttcactcctcccc
B tattgaactccaccccactcctcccctcctctcctccactcctccccttcactcctcccc
B tattgaactccaccccactcctcccctcctctcctccactcctccccttcactcctcccc
J tattgaaccaagccactcctctc--ctccattcctgccctcctctcctccactcctcccc
J tattgaaccaagccactcctctc--ctccattcctgccctcctctcctccactcctcccc
G tattgaaccaagccactcctctccactcctcccctccactcctctccttctctcctcccc
N tattgaactccaccccactcctcccttcctctccttcactcctcccctcctctcctccac
C tattgaactccaccccactcctcccctcctctcctccactcctccccttcactcctcccc

M ------------------------------------------------------------
M ------------------------------------------------------------
M ------------------------------------------------------------
M ------------------------------------------------------------
M ------------------------------------------------------------
L ------------------------------------------------------------
L ------------------------------------------------------------
L ------------------------------------------------------------
K ------------------------------------------------------------
I -------------------------------------------------ctccttc---t
```

```
D -------------------------------------------------ctccttc---t
E -------------------------------------------------ctccttc---t
F tc---------------------------------------------ccctcctcc---a
H tc---------------------------------------------ctctcctcc---a
B tc---------------------------------------------ctctcctcc---c
B tc---------------------------------------------ctctcctcc---c
J tc---------------------------------------------ctctcctccactc
J tc---------------------------------------------ctctcctccactc
G tcctccactcctcccctcctctcctcctctcctccccttctctcctcccctcctcc---a
N cc---------------------------------------------cactcctcc---c
C tc---------------------------------------------ctctcctcccctc

M ------------------------------------------------------------
M ------------------------------------------------------------
M ------------------------------------------------------------
M ------------------------------------------------------------
M ------------------------------------------------------------
L ------------------------------------------------------------
L ------------------------------------------------------------
L ------------------------------------------------------------
K ------------------------------------------------------------
I ctcctcc-----------------------------------------------------
D ctcctcc-----------------------------------------------------
E ctcctcc-----------------------------------------------------
F ctcctcc-----------------------------------------------------
H ctcctct-----------------------------------------------------
B ctcctctcctc-------------------------------------------------
B ctcctctcctc-------------------------------------------------
J ctcccctcctc-------------------------------------------------
J ctcccctcctc-------------------------------------------------
G ctcctcc-----------------------------------------------------
N ctcctctcctc-------------------------------------------------
C ctctcctcctcaaagtcaatactttgagttttcctaattagagggaaagattcacagtgt
```

<The 5940bp sequences of intron is omitted here>

```
M ------------------------------------------------------------
M ------------------------------------------------------------
M ------------------------------------------------------------
M ------------------------------------------------------------
M ------------------------------------------------------------
L ------------------------------------------------------------
L ------------------------------------------------------------
L ------------------------------------------------------------
K ------------------------------------------------------------
```

```
I ----------------------------------------tcctccactcctgc------
D ----------------------------------------tcctccactcctgc------
E ----------------------------------------tcctccactcctgc------
F ----------------------------------------tcctccactcctgc------
H ----------------------------------------tcctccactcctgc------
B ----------------------------------------tcctccactcctccactcct
B ----------------------------------------tcctccactcctccactcct
J ----------------------------------------tcctccactcctccgct---
J ----------------------------------------tcctccactcctccgct---
G ----------------------------------------tcctccactcctgc------
N ------------------------------------------tcctcctcctccactcct
C acttctctgagggagcaaggggccagccagctcaagtccagcctagccttgcctaggggc

M ----------------------ctctcctcccctcccctttactcctctcctccttcact
M ----------------------ctctcctcccctcccctttactcctctcctccttcact
M ----------------------ctctcctcccctcccctttactcctctcctccttcact
M ----------------------ctctcctcccctcccctttactcctctcctccttcact
M ----------------------ctctcctcccctcccctttactcctctcctccttcact
L ----------------------ctctcctcccctcccctttactcctctcctccttcact
L ----------------------ctctcctcccctcccctttactcctctcctccttcact
L ----------------------ctctcctcccctcccctttactcctctcctccttcact
K ----------------------ctctcctcccctcccctttactcctctcctccttcact
I -----cctccacttctcccctcctctcctcccctcccctttactcctctcctccttcact
D -----cctccacttctcccctcctctcctcccctcccctttactcctctcctccttcact
E -----cctccacttctcccctcctctcctcccctcccctttattcctctcctccttcact
F -----cctccacttctccc-----------------------ctcctctcctccttcact
H -----cctccacttctcccctcctctcctcccctcccctttactcctctcctccttcact
B cccctcctcccctcctctcctccactcctcccctcccctttactcctctcctccttcact
B cccctcctcccctcctctcctccactcctcccctcccctttactcctctcctccttcact
J ---------------------------cctcccccccctttattcctctcctccttcact
J ---------------------------cctcccccccctttattcctctcctccttcact
G -----cctccacttctcccctcctctcctcccctcccctttactcctctcctccttcact
N ccactcatcccctcctctcctccactcctcccctcccctttactcctctcctccttcact
C cagcccctcccctcctctcctccactcctcccctcccctttactcctctcctccttcact

[EXON 2]
M ctc---------tgctttcagacttataaacgagtacatgaaaaaaggagaccccatcct
M ctc---------tgctttcagacttataaacgagtacatgaaaaaaggagaccccatcct
M ctc---------tgctttcagacttataaacgagtacatgaaaaaaggagaccccatcct
M ctc---------tgctttcagacttataaacgagtacatgaaaaaaggagaccccatcct
M ctc---------tgctttcagacttataaacgagtacatgaaaaaaggagaccccatcct
L ctc---------tgctttcagacttagaaacgagtacatgaaaaaaggagaccccatcct
L ctc---------tgctttcagacttagaaacgagtacatgaaaaaaggagaccccatcct
L ctc---------tgctttcagacttagaaacgagtacatgaaaaaaggagaccccatcct
```

```
K ctc---------tgctttcagacttataaacgagtacatgaaaaaaggagaccccatcct
I ctc---------tgctttcagatttataaacgagtacatg-aagaaggagaccccatcct
D ctc---------tgctttcagatttataaacgagtacatg-aagaaggagaccccatcct
E ctctactttcagtgctttcagatttataaacgagtacatg-aagaaggagaccccatcct
F ctc---------tgctttcagatttataaacgagtacatg-aagaaggagaccccatcct
H ctc---------tgctttcagatttataaacgagtacatg-aagaaggagaccccatcct
B ctctactttcagtgctttcagatttagaaatgagtacatgaaaaaaggagaccccatcct
B ctctactttcagtgctttcagatttagaaatgagtacatgaaaaaaggagaccccatcct
J ctctactttcagtgctttcagatttcgaaacgagtacatgaaaaaaggagaccccagcct
J ctctactttcagtgctttcagatttcgaaacgagtacatgaaaaaaggagaccccagcct
G ctc---------tgctttcagatttataaacgagtacatg-aagaaggagaccccatcct
N ctctactttcagcgctttcagacttagaaatgagtacatg-aagaaggagaccccatcct
C ctctactttcagtgctttcagatttagaaatgagtacatgaaaaaaggagaccccatcct
  ***         .*********.**  ***.********* **.************ ***

M tccctcctcaaggacagctggaggatcaggggactaaggtccatcaaactatcatttca
M tccctcctcaaggacagctggaggatcaggggactaaggtccatcaaactatcatttca
M tccctcctcaaggacagctggaggatcaggggactaaggtccatcaaactatcatttca
M tccctcctcaaggacagctggaggatcaggggactaaggtccatcaaactatcatttca
M tccctcctcaaggacagctggaggatcaggggactaaggtccatcaaactatcatttca
L tccctcctcaaggacagctggaggatcaggggactaaggtccatcaaactatcatttca
L tccctcctcaaggacagctggaggatcaggggactaaggtccatcaaactatcatttca
L tccctcctcaaggacagctggaggatcaggggactaaggtccatcaaactatcatttca
K tccctcctcaaggacagctggaggatcaggggactaaggtccatcaaactatcatttca
I tccctcctcaaggacagctggaggatcaggggattaaggtccatcaaactatcatttca
D tccctcctcaaggacagctggaggatcaggggattaaggtccatcaaactatcatttca
E tccctcctcaaggacagctggaggatcaggggactaaggtccatcaaactatcatttca
F tccctcctcaaggacagctggaggatcaggggactaaggtccatcaaactatcatttca
H tccctcctcaaggacagctggaggatcaggggactaaggtccatcaaactatcatttca
B tccctcctcaaggacagctggggatcaggggactaaggtccatcaaactatcatttca
B tccctcctcaaggacagctggggatcaggggactaaggtccatcaaactatcatttca
J tctctcctcaaggacagctggaggatcaggggactaaggtccatcaaactatcatttca
J tctctcctcaaggacagctggaggatcaggggactaaggtccatcaaactatcatttca
G tccctcctcaaggacagctggaggatcaggggactaaggtccatcaaactatcatttca
N tccctcctcaaggacagctggaggatcaggggactaaggtccatcaaactatcatttca
C tccctcctcaaggacagctggggatcaggggactaaggtccatcaaactatcatttca
  **.******************.************.*************************

M ctcctcccagagggtcgagatgccccaccagtcaggtgcccagtgctggag---------
M ctcctcccagagggtcgagatgccccaccagtcaggtgcccagtgctggag---------
M ctcctcccagagggtcgagatgccccaccagtcaggtgcccagtgctggag---------
M ctcctcccagagggtcgagatgccccaccagtcaggtgcccagtgctggag---------
M ctcctcccagagggtcgagatgccccaccagtcaggtgcccagtgctggag---------
L ctcctcccggagggtcgagatgccccaccagtcaggtgcccagtgctggagaggaccccc
```

```
L ctcctcccggagggtcgagatgccccaccagtcaggtgcccagtgctggagaggaccccc
L ctcctcccggagggtcgagatgccccaccagtcaggtgcccagtgctggagaggaccccc
K ctcctcccagaggatcgagatgccccaccagtcaggtgcccagtgctggagaggaccccc
I ctcctcccagaggatcgagatgccccaccagtcaggtgcccagtgctggagaggaccccc
D ctcctcccagaggatcgagatgccccaccagtcaggtgcccagtgctggagaggaccccc
E ctcctcccagaggatcgagatgccccaccagtcaggtgcccagtgctggagaggaccccc
F ctcctcccagaggatcgagatgccccaccagtcaggtgcccagtgctggagaggaccccc
H ctcctcccagaggatcgagatgccccaccagtcaggtgcccagtgctggagaggaccccc
B ctcctcccagaggatcgagatgccccaccagtcagttgcccagtgctggagaggaccccc
B ctcctcccagaggatcgagatgccccaccagtcagttgcccagtgctggagaggaccccc
J ctcctcccagaggatcgagataccccaccagtcaggtgcccagtgctggagaggaccccc
J ctcctcccagaggatcgagataccccaccagtcaggtgcccagtgctggagaggaccccc
G ctcctcccagaggatcgagatgccccaccagtcaggtgcccagtgctggagaggaccccc
N ctcctcccagaggatcgagatgccccaccagtcaggtgcccagtgctggagaggaccccc
C ctcctcccagaggatcgagatgccccaccagtcagttgcccagtgctggagaggaccccc
  ********.****.*******.************* ***************

M aggacccccaggtgtatccgatggcagacagctgcgtctgtcgaacgggagttcagatta
M aggacccccaggtgtatccgatggcagacagctgcgtctgtcgaacgggagttcagatta
M aggacccccaggtgtatccgatggcagacagctgcgtctgtcgaacgggagttcagatta
M aggacccccaggtgtatccgatggcagacagctgcgtctgtcgaacgggagttcagatta
M aggacccccaggtgtatccgatggcagacagctgcgtctgtcgaacgggagttcagatta
L aggacccccaggtgtatccgatggcagacagctgcgtctgtcgaacgggatttcagatta
L aggacccccaggtgtatccgatggcagacagctgcgtctgtcgaacgggatttcagatta
L aggacccccaggtgtatccgatggcagacagctgcgtctgtcgaacgggatttcagatta
K aggacccccaggtgtatccgatggcagacagctgcgtctgtcgaacgggagttcagatta
I aggacccccaggtgtatccgatggcagacagctgcgtctgtcgaaggggagttcagatta
D aggacccccaggtgtatccgatggcagacagctgcgtctgtcgaaggggagttcagatta
E aggacccccaggtgtatccgatggcagacagctgcgtctgtcgaaggggagttcagatta
F aggacccccaggtgtatccgatggcagacagctgcgtctgtcgaaggggagttcagatta
H aggacccccaggtgtatccgatggcagacagctgcgtctgtcgaaggggagttcagatta
B aggacccccaggtgtatccgatggcagacagcggcgtctgtcgaaggggagttcagattt
B aggacccccaggtgtatccgatggcagacagcggcgtctgtcgaaggggagttcagattt
J aggacccccaggtgtatccgatggcagacagctgcgtctgtcgaaggggagttcagattt
J aggacccccaggtgtatccgatggcagacagctgcgtctgtcgaaggggagttcagattt
G aggacccccaggtgtatc------------------------------------------
N aggacccccaggtgtatccgatggcagacagctgcgtctgtcgaaggggagttcagatta
C aggacccccaggtgtatccgatggcagacagctgcgtctgtcgaaggggagttcagattt
  ******************

M gattagattaacccaaacccagcaccctcacaac--tgtattgtgcttgtatcatttagg
M gattagattaacccaaacccagcaccctcacaac--tgtattgtgcttgtatcatttagg
M gattagattaacccaaacccagcaccctcacaac--tgtattgtgcttgtatcatttagg
M gattagattaacccaaacccagcaccctcacaac--tgtattgtgcttgtatcatttagg
```

```
M gattagattaacccaaacccagcaccctcacaac--tgtattgtgcttgtatcatttagg
L gattagattaacccaaacccagcaccctcacaac--tgtattgtgcttgtatcatttagg
L gattagattaacccaaacccagcaccctcacaac--tgtattgtgcttgtatcatttagg
L gattagattaacccaaacccagcaccctcacaac--tgtattgtgcttgtatcatttagg
K gattagattaacccaaacccagcaccctcacaac--tgtattgtgcttgtatcatttagg
I gattagattaacctaaacccagcaccctcacaactatgtattgtgccagtatcattttgg
D gattagattaacctaaacccagcaccctcacaactatgtattgtgccagtatcattttgg
E gattagattaacctaaacccagcaccctcacaactatgtattgtgccagtatcattttgg
F gattagattaacctaaacccagcaccctcacaactatgtattgtgccagtatcattttgg
H gattagattaacctaaacccagcaccctcacaactatgtattgtgccagtatcattttgg
B gatttgattaacccaaacccagcagcctcacaactatgtattgtgcctgtatcattttgg
B gatttgattaacccaaacccagcagcctcacaactatgtattgtgcctgtatcattttgg
J gatttgattaacccaaacccagcagcctcacaactatgtattgtgcctgtatcattttgg
J gatttgattaacccaaacccagcagcctcacaactatgtattgtgcctgtatcattttgg
G ------------------------------------------------------------
N gattagattaacctaaacccagcaccctcacaactatgtattgtgccagtatcattttgg
C gatttgattaacccaaacccagcagcctcacaactatgtattgtgcctgtatcattttgg

M attttcaaagcagcaactgatttgagttgatcaagttgttcac
M attttcaaagcagcaactgatttgagttgatcaagttgttcac
M attttcaaagcagcaactgatttgagttgatcaagttgttcac
M attttcaaagcagcaactgatttgagttgatcaagttgttcac
M attttcaaagcagcaactgatttgagttgatcaagttgttcac
L attttcaaagcaacaactgatttgagttgatcaagttgttcac
L attttcaaagcaacaactgatttgagttgatcaagttgttcac
L attttcaaagcaacaactgatttgagttgatcaagttgttcac
K attttcaaagcaacaatttatttgagttgatcaagttgttcac
I attttcaaagcaacaactgatttgagttgatcaagttgttcac
D attttcaaagcaacaactgatttgagttgatcaagttgttcac
E attttcaaagcaacaactgatttgagttgatcaagttgttcac
F attttcaaagcaacaactgatttgagttgatcaagttgttcac
H attttcaaagcaacaactgatttgagttgatcaagttgttcac
B --------------aactgatttgagttgatcaagttgttcac
B --------------aactgatttgagttgatcaagttgttcac
J --------------aactgatttgagttgatcaagttgttcac
J --------------aactgatttgagttgatcaagttgttcac
G -------------------------------------------
N attttcaaagcaacaactgatttgagttgatcaagttgttcac
C --------------aactgatttgagttgatcaagttgttcac
```

TRANSPARENT METHODS

Sample collection and identification.

The methods were carried out in accordance with the approved guidelines of the Good Experimental Practices adopted by the Institute of Zoology, Chinese Academy of Sciences (CAS). All procedures described in this study were approved by the Committee for Animal Experiments at the Institute of Zoology, Chinese Academy of Sciences. An adult male *P. chinensis* was collected from the Guanting Reservoir in Hebei province for de novo sequencing. The specimen was confirmed to be *P. chinensis* by DNA barcoding analysis [as outlined by (Ward et al., 2005)]. Specimens for transcriptome (RNA-seq) sequencing were collected from Hongze Lake, Jiangsu province. We followed the method of Kimmel (Kimmel et al., 1995) to define four development stages: the pharyngula stage, the hatching stage, larval fish stage, and the adult stage.

Genome sequencing and assembly

Genomic DNA was extracted from a whole-animal using a DNeasy Blood & Tissue Kit (QIAGEN). To sequence the *P. chinensis* genome, we employed PacBio Sequel long-read sequencing and 10X Genomics Chromium linked reads sequencing, coupled with short-read sequencing of 250bp, 350bp, 2kb, 5kb, 10kb paired-end libraries on the Illumina platform (Table S1). Pacific Biosciences SMRTbell libraries were prepared using 10 kb and 20 kb preparation protocols. The main steps for library preparation were: (1) gDNA shearing; (2) DNA damage repair; (3) blunt end-ligation with hairpin adapters from the SMRTbell Template Prep Kit 1.0 (Pacific Biosciences); (4) size selection; and (5) binding to polymerase. Sequencing was performed on a PacBio Sequel instrument with Sequel Sequencing Kit 1.2.1. A total of 10.57 Gb (21.83×) PacBio reads were generated. 10X Genomics Chromium sequencing (also known as

the Chromium Genome Solution) allows long-range sequence information to be generated on a short-read Illumina sequencer by barcoding long DNA molecules before preparation of short-read fragment DNA libraries (Zheng et al., 2016). The barcodes (also known as linked-reads) can be used to obtain 'synthetic long reads'. A total of 81.92 Gb (169.22×) 10X Genomics linked-reads were generated. The initial assembly was generated using Allpaths-LG v44080 (Butler et al., 2008) and 250 bp, 350 bp, 2 kb, 5 kb, and 10 kb Illumina data, followed by PacBio data and gap filling using PBJelly v14.1 (English et al., 2012) and two-rounds of polishing using Pilon v1.18 (Walker et al., 2014) and Illumina reads. Finally, 10X Genomics linked-reads were used to link scaffolds using fragScaff v140324 (Adey et al., 2014). Genome completeness was assessed by mapping de novo assembled transcripts to the genome (see below), and by CEGMA v2.5 (Parra et al., 2007) and BUSCO v1.1 (Waterhouse et al., 2017) evolutionary conserved gene set analysis.

Experimental model and subject details

A broodstock of *P. chinensis* was collected from Hongze Lake, Jiangsu province. After artificial insemination, eggs were transported (at 4-10°C) to the Chinese Academy of Sciences Institute of Zoology in Beijing for incubation experiments on 7 Jan 2017. The eggs were hatched in an experimental glass tank (200-300 eggs per 3L tank), with a 12:12-hour light:dark regime. The water temperature was 10-15°C. Water changes were performed daily (70% exchanged).

Transcriptome sequencing

Total RNAs was isolated from four different development stages: the pharyngula stage, the hatching stage, larval fish stage, and the adult stage. RNA sequencing libraries were constructed using the Illumina

mRNA-Seq Prep Kit. Briefly, oligo(dT) magnetic beads were used to mRNA molecules. Paired-end libraries were sequenced on the Illumina HiSeq platform, and 150 bp paired-end reads were generated. Raw sequencing reads were filtered for base quality >15 and read length >30 bp using the Novogene-developed application ng_QC v2.0 with default parameters (i.e., L:5 -p:0.5 -N:0.1). We used TopHat v1.3.1 (Trapnell et al., 2009) to align RNA-seq reads to the genome. Gene expression was quantified as reads per kilobase of gene per million mapped reads (RPKM). RPKM values were scaled using the TMM (trimmed mean of M values; M values mean the log expression ratios) method (Robinson and Oshlack, 2010). A *P. chinensis* transcriptome was also generated (*de novo* assembled) from pooled RNA-seq samples using Trinity v2.1.1 (Haas et al., 2013) with the parameters '-ss 0.5 -jc 0 -minkmercov 2 -minglue 2'.

Estimation of genome size using *k*-mer method

Genome size can be estimated by *k*-mer frequency analysis (Liu et al., 2013). Error-corrected [NGS QC Toolkit v2.3.3 (Patel et al., 2012)] 180 bp to 270 bp Illumina genome sequencing reads (~229.86 Gb data) were used to estimate the genome size of *P. chinensis*. The distribution of 17 *k*-mers showed a major peak at 78-fold depth (Table S2; Figure S1A). Based on the total number of reads (38,879,079,724) and corresponding to a *k*-mer depth of 78, the *P. chinensis* genome size was estimated to be ~484.10Mbp using the formula ‘Genome size= kmer_Number/Peak_Depth’.

Genome assembly assessment

The *P. chinensis* assembly was evaluated by mapping Illumina short-insert library genome sequencing reads (see the section above) to the assembly using BWA v0.7.8 (Li and Durbin, 2010) (Table S5; Figure

S1B). De novo transcriptome reads were mapped to the assembly using BLAT v0.35 (Kent, 2002)(Table S8). We also employed two methods which employ core gene sets to assess genome completeness (Table S9 and S10): CEGMA v2.5 (Core Eukaryotic Genes Mapping Approach) (Parra et al., 2007) compares a set of 248 core eukaryotic genes to an assembled genome, while BUSCO v1.1 (Benchmarking Universal Single-Copy Orthologs) (Seppey et al., 2019; Simao et al., 2015) compares near-universal single-copy orthologs. To assess GC bias, we plotted the distribution of GC content against sequencing depth (Figure S1C and S1D).

Genome annotation

Repeats, including repetitive sequences and transposable elements, were identified using RepeatMasker v4.0.5 (Tarailo-Graovac and Chen, 2009) and either the RepBase vertebrate library (Bao et al., 2015) or a de novo repeat library [built using RepeatModeler] (Figure S1E). Tandem repeats were identified by searching for two or more contiguous, approximate copies of a pattern of nucleotides using Tandem Repeats Finder v407 (Benson, 1999).

Homology-based predictions, de novo predictions, and transcriptome-based prediction methods were used to annotate the protein-coding genes of *P. chinensis*. For homology-based gene prediction, protein sequences from nine other sequenced teleost genomes [*Danio rerio* (zebrafish), *Tetraodon nigroviridis* (pufferfish), *Gasterosteus aculeatus* (stickleback), *Oryzias latipes* (medaka), *Salmo salar* (salmon), *Cynoglossus semilaevis* (flatfish), *Takifugu rubripes* (fugu), *Oreochromis niloticus* (tilapia), and *Larimichthys crocea* (yellow croaker)] were used to query the *P. chinensis* genome using tBLASTn v2.2.26 (E-value $\leq 10^{-5}$) (Camacho et al., 2009). Next, the homologous genome sequences were aligned against the matching proteins using GeneWise V2.4.1(Birney et al., 2004) to take into account splice site

variation. Three de novo gene prediction tools Augustus v3.1 (Stanke et al., 2006), GlimmerHMM v3.0.4 (Majoros et al., 2004), and SNAP (Korf, 2004) were employed to predict genes in the repeat-masked *P. chinensis* genome. RNA-seq reads from *P. chinensis* [whole-fish from four development stages Given the small size of P. chinensis, several individuals were pooled for each sample type] were aligned to the genome using TopHat v2.0.11 (Trapnell et al., 2009) and Cufflinks v2.1.1 (Trapnell et al., 2014) was used to produce assembled transcripts and predict transcript structures. Data from the three prediction methods were merged into CDS models using EVM v1.1.1 (Haas et al., 2008), and untranslated (UTR) and isoforms were constructed using PASA v2.0.2 (Haas et al., 2003).

We next performed functional annotation of protein-coding genes in the *P. chinensis* genome. The predicted protein sequences of *P. chinensis* were assessed using publicly available databases – Swiss-Prot (Artimo et al., 2012), NR (non-redundant nucleotides) (O'Leary et al., 2016), KEGG (Kanehisa et al., 2017), and InterPro (Zdobnov and Apweiler, 2001) using BLASTp (Camacho et al., 2009) (*E*-value $\leq 10^{-5}$) – and the best hit for each query retained. For each gene, its Gene Ontology (GO) term(s) and Pfam accession were used to query various additional databases (ProDom, HAMAP, PANTHER, TIGRFAMs, PRINTS, PIRSF, Gene3D, COILS, PROSITE, Pfam, and SMART) (Attwood et al., 2003; Corpet et al., 2000; Falquet et al., 2002; Haft et al., 2003; Lees et al., 2014; Lupas et al., 1991; Punta et al., 2012; Schultz et al., 1998; Tania et al., 2009; Thomas et al., 2003; Wu et al., 2004). Non-coding RNA genes were also identified. The tRNAscan-SE (Lowe and Eddy, 1997) software (v1.3.1) was used to predict tRNA sequences. We aligned the *P. chinensis* genome to the rRNA sequences of *Homo sapiens* using BLASTn (*E*-value $\leq 10^{-5}$) (Camacho et al., 2009). The miRNA and snRNA genes of *P. chinensis* were extracted using v1.1rc4 Infernal (Nawrocki and Eddy, 2013) and against the Rfam database (Griffiths-Jones et al., 2005).

Orthology and phylogenomics

A total of 18 fish species, including *P. chinensis*, were selected for orthology analysis. Orthology was determined using the OrthoMCL (Li et al., 2003) pipeline. Briefly, we first filtered out redundant splice variants – retaining the longest isoform of each protein set – followed by all-against-all protein comparisons using BLASTp (Camacho et al., 2009) (E-value $\leq 10^{-5}$). High-scoring Segment Pair (HSPs) were processed by MCL v10-201 (Enright et al., 2002) to define orthologs, inparalogs, and co-orthologs. Alignments with high-scoring segment pairs (HSPs) were conjoined for each gene pair using SOLAR (Sorting Out Local Alignment Results). More than 30% coverage of the aligned region in both homologous genes was required to assign homologous gene-pairs.

To generate a phylogenetic tree, 627 single-copy ortholog nucleotide alignments (coding sequence; CDS) from 18 species (*P. chinensis*, *Gasterosteus aculeatus*, *Danio rerio*, *Lepisosteus oculatus*, *Gadus morhu*, *Takifugu rubripes*, *Nothobranchius furzeri*, *Esox lucius*, *Oreochromis niloticus*, *Xiphophorus maculatus*, *Oryzias latipes*, *Salmo salar*, *Scleropages formosus*, *Anguilla rostrata*, *Latimeria chalumnae*, *Hippocampus comes*, *Ictalurus punctatus*, and *Callorhinchus milii*) were concatenated into a super-alignment. Multiple alignments of coding sequences (CDS) for each ortholog group were performed using MUSCLE v3.7 (Edgar, 2004). jModelTest v2.1.2 was used to select the best substitution model by Akaike information criterion (AIC). The species tree was obtained using RAxML v704 (Stamatakis, 2014) and the GTR+GAMMA model, with 100 replicates of bootstrap analysis. Species divergence times were inferred using MCMCTree (Donoghue et al., 2009), included in PAML v4.7a (Yang, 2007), with the parameters 'RootAge = <500 model = REV (GTR) alpha = 0.666853 clock = 3', and the calibration points as prior [obtained from (Benson, 1999; Bian et al., 2016; Schartl et al.,

2013; Yang et al., 2016)] are provided in (Table S14).

Expansion and contraction of gene families

We determined the expansion and contraction of gene families by comparing the cluster size differences between the of the *P. chinensis* and 17 other fish species using CAFE (Version 1.6) (De Bie et al., 2006). A random birth and death model was used to study changes of gene families along each lineage of phylogenetic tree. A probabilistic graphical model (PGM) was introduced to calculate the probability of transitions in gene family size from parent to child nodes in the phylogeny. Using conditional likelihoods as the test statistics, we calculated the corresponding *P*-values in each lineage. A *P*-value of 0.05 was used to denote families significantly expanded in the *P. chinensis* genome.

Identification of single-copy gene families gained by *P. chinensis*

We clustered paralogs and orthologs using the OrthoMCL method (Li et al., 2003) (BLASTp *E*-value ≤ 10^{-5}) and 18 sequenced fish species (*P. chinensis*, *Gasterosteus aculeatus*, *Danio rerio*, *Lepisosteus oculatus*, *Gadus morhu*, *Takifugu rubripes*, *Nothobranchius furzeri*, *Esox lucius*, *Oreochromis niloticus*, *Xiphophorus maculatus*, *Oryzias latipes*, *Salmo salar*, *Scleropages formosus*, *Anguilla rostrata*, *Latimeria chalumnae*, *Hippocampus comes*, *Ictalurus punctatus*, and *Callorhinchus milii*).

Identification of positively selected genes

Positive selection on an ORF-wide level was estimated using in-frame codon alignments and the Branch-site Unrestricted Statistical Test for Episodic Diversification (BUSTED) method implemented in HyPhy v2.5.9 (Pond et al., 2005). BUSTED requires a prior partitioning of branches into foreground and

background branches and considered to more accurately identify episodic (acting only on particular lineages) positive selection (Murrell et al., 2015; Spielman et al., 2019). In the species tree, the *P. chinensis* lineage was marked as 'foreground' and the rest of the fish species (*Gadus morhua*, *Gasterosteus aculeatus*, *Danio rerio*, *Oreochromis niloticus*, *Esox lucius*, *Oryzias latipes*, *Xiphophorus maculatus*, and *Scleropages formosus*) as 'background'.

Prediction of bone and scale genes

Genes involved in vertebrate bone formation were obtained from (Venkatesh et al., 2014). If a gene could not be found by searching *P. chinensis* gene names and symbols, we obtained the gene (CDS and protein sequence) from a dataset of *O. latipes*, *G. aculeatus*, *T. rubripes*, *E. Lucius*, *I. punctatus*, and *D. rerio* and queried the *P. chinensis* genome using BLAST (Camacho et al., 2009) or GeneWise (Birney et al., 2004) [using protein sequences as query]. Predictions were also made using *ab initio* methods, such as FGENESH (Solovyev et al., 2006), when no *P. chinensis* sequence could be obtained. All predictions were manually curated.

Staining of the *P. chinensis* skeleton

Adult bones and cartilage were stained with Alizarin red and Alcian blue, respectively. Briefly, a fish specimen was fixed in formalin (10% formaldehyde), briefly dehydrated in 70% ethanol, decolorized with 3% hydrogen peroxide for 6 hours, and placed in Alcian blue for 12 hours. The specimen was dehydrated using 50% ethanol for 48 hours, and bones were next stained with 2 g/l Alizarin red and detained with 1% KOH until background stain was lost.

Hox gene analysis

Hox genes from zebrafish, as well as Atlantic salmon (*Salmo salar*) and Northern pike (*Esox Lucius*) were used to query the *P. chinensis* genome using GeneWise v2.4.1 (Birney et al., 2004). *Hox* gene clusters were next manually curated. We performed PCR and Sanger sequencing of the following *P. chinensis* pseudogenes to rule out genome sequencing or assembly errors: *HOXB3b* (5'-AGAGATTGACAGGGGCATGG-3' and 5'-TGATAGATGTAGGTCCACTGTTG-3'; T_a=56 °C), *HOXB8b* (5'-CCTAAGTGTATCTAAAACGT-3 and 5'-ATTCTACATTCTACATTTCC-3; T_a=55 °C), *HOXC3a* (5'-CCACACAGACATTTAGAGGC-3 and 5'-TAAGGGCATAATCCAGTCGA-3; T_a=55 °C), and *HOXC5a* (5'-CCTGGATTATTTGGGGCAGG-3 and 5'-TGAAATTCACAACCGTTCAACA-3; T_a=58 °C).

Sequencing and analysis of _P. chinensis_ fibroblast growth factor 5 genes

P. chinensis FGF5-derived genes were amplified from whole-fish genomic DNA using PrimeSTAR HS DNA Polymerase (TaKaRa) – with a forward primer in exon 1 and a reverse primer in an exon unique to a novel *P. chinensis FGF5* exon (5'-GTTCCTCTTTGTCTTTATACCGTC-3' and 5'-GTGAACAACTTGATCAACTCAAATC-3', T_a=52 °C) – on a ABI-9700 (ABI) thermal cycler and Sanger sequenced. The MAFFT online server v7.452 (http://mafft.cbrc.jp) (Katoh et al., 2019; Kuraku et al., 2013) was used to generate multiple sequence alignments of amplicons and *FGF5* genome scaffolds (using the G-INS-i Iterative refinement method). The settings for local instances of MAFFT are 'mafft --threadtb 5 --threadit 0 --reorder --leavegappyregion --maxiterate 1000 --retree 1 --globalpair') and a phylogenetic tree [neighbor joining tree generated from conserved sites, maximum likelihood (ML) analysis was generated using RaxML v8 (Stamatakis, 2014) with the search strategy set to rapid

bootstrapping and 1,000 bootstrap replicates]. The deduced amino acid sequences were predicted using the ExPASy translate tool (https://web.expasy.org/translate) (Artimo et al., 2012). In addition, the *FGF5* phylogenetic tree was generated from *FGF5* protein sequences of *P. chinensis* and other 10 species (*Scleropages Formosus*, *Letalurus punctatus*, *Danio rerio*, *Sinocyclocheilus anshuiensis*, *Esox lucius*, *Salmo salar*, *Xiphophorus maculatus*, *Larimichthys crocea*, *Oryzias latipes*, *Takifugu rubripes*). The maximum likelihood (ML) analysis was generated using RaxML v8 (Stamatakis, 2014) with the ProtCAT model search strategy set to rapid bootstrapping and 1,000 bootstrap replicates. Protein sequences were aligned using MAFFT with default parameter.

Immune system analysis

We retrieved immunity-related genes in the *P. chinensis* from our annotation pipeline as well as by manual curation. Sequence alignments were obtained using ClustalX v2.1 (Larkin et al., 2007). A neighbor-joining phylogenetic tree of the TLR gene family was conducted from multiple sequence alignments of proteins using MAGE6 (Tamura et al., 2013).

Assessment of pigmentation genes

Pigmentation genes of interest in *P. chinensis* were interrogated by BLAST (Camacho et al., 2009) analysis of the genome assembly and whole-fish RNA-seq data (raw reads and Trinity assembly) on a local instance of sequenceserver v1.1.0 (Priyam et al., 2015), using zebrafish gene sequences as the query. Regions with unique changes in *P. chinensis* were next investigated by BLAST searches against the NCBI databases RefSeq (curated genomes, transcripts, and proteins) and NR (O'Leary et al., 2016), and the ~15,000-proteome database UniProt (The UniProt, 2017) [the number of output alignments was set

to 1,000]. Obtained sequences were aligned with the *P. chinensis* query using the MAFFT web server (Katoh et al., 2019). The impact of amino acid residue changes on protein function, structure, and stability was assessed using the online tools PANTHER-PSEP (Tang and Thomas, 2016), PolyPhen2 (Adzhubei et al., 2010), SIFT (Kumar et al., 2009), and I-Mutant 2.0 (Capriotti et al., 2005).

Quantification and statistical analyses

Statistics details are provided in the Methods Details section.

SUPPLEMENTAL REFERENCES

Adey, A., Kitzman, J.O., Burton, J.N., Daza, R., Kumar, A., Christiansen, L., Ronaghi, M., Amini, S., Gunderson, K.L., Steemers, F.J., *et al.* (2014). In vitro, long-range sequence information for de novo genome assembly via transposase contiguity. Genome Res *24*, 2041-2049.

Adzhubei, I.A., Schmidt, S., Peshkin, L., Ramensky, V.E., Gerasimova, A., Bork, P., Kondrashov, A.S., and Sunyaev, S.R. (2010). A method and server for predicting damaging missense mutations. Nat Methods *7*, 248-249.

Artimo, P., Jonnalagedda, M., Arnold, K., Baratin, D., Csardi, G., de Castro, E., Duvaud, S., Flegel, V., Fortier, A., Gasteiger, E., *et al.* (2012). ExPASy: SIB bioinformatics resource portal. Nucleic Acids Res *40*, W597-603.

Attwood, T.K., Bradley, P., Flower, D.R., Gaulton, A., Maudling, N., Mitchell, A.L., Moulton, G., Nordle, A., Paine, K., Taylor, P., *et al.* (2003). PRINTS and its automatic supplement, prePRINTS. Nucleic Acids Research *31*, 400-402.

Bao, W.D., Kojima, K.K., and Kohany, O. (2015). Repbase Update, a database of repetitive elements in eukaryotic genomes. Mobile DNA-Uk *6*.

Benson, G. (1999). Tandem repeats finder: a program to analyze DNA sequences. Nucleic Acids Research *27*, 573-580.

Bian, C., Hu, Y., Ravi, V., Kuznetsova, I.S., Shen, X., Mu, X., Sun, Y., You, X., Li, J., Li, X., *et al.* (2016). The Asian arowana (Scleropages formosus) genome provides new insights into the evolution of an early lineage of teleosts. Sci Rep *6*, 24501.

Birney, E., Clamp, M., and Durbin, R. (2004). GeneWise and genomewise. Genome Research *14*, 988-995.

Butler, J., MacCallum, I., Kleber, M., Shlyakhter, I.A., Belmonte, M.K., Lander, E.S., Nusbaum, C., and Jaffe, D.B. (2008). ALLPATHS: de novo assembly of whole-genome shotgun microreads. Genome Research *18*, 810-820.

Camacho, C., Coulouris, G., Avagyan, V., Ma, N., Papadopoulos, J., Bealer, K., and Madden, T.L. (2009). BLAST+: architecture and applications. BMC Bioinformatics *10*, 421.

Capriotti, E., Fariselli, P., and Casadio, R. (2005). I-Mutant2.0: predicting stability changes upon mutation from the protein sequence or structure. Nucleic Acids Res *33*, W306-310.

Corpet, F., Servant, F., Gouzy, J., and Kahn, D. (2000). ProDom and ProDom-CG: tools for protein domain analysis and whole genome comparisons. Nucleic Acids Research *28*, 267-269.

De Bie, T., Cristianini, N., Demuth, J.P., and Hahn, M.W. (2006). CAFE: a computational tool for the study of gene family evolution. Bioinformatics *22*, 1269-1271.

Donoghue, P., Benton, M., Yang, Z.H., and Inoue, J. (2009). Calibrating and Constraining the Molecular Clock. J Vertebr Paleontol *29*, 89a-89a.

Edgar, R.C. (2004). MUSCLE: multiple sequence alignment with high accuracy and high throughput. Nucleic Acids Research *32*, 1792-1797.

English, A.C., Richards, S., Han, Y., Wang, M., Vee, V., Qu, J.X., Qin, X., Muzny, D.M., Reid, J.G., Worley, K.C.*, et al.* (2012). Mind the Gap: Upgrading Genomes with Pacific Biosciences RS Long-Read Sequencing Technology. PloS one *7*.

Enright, A.J., Van Dongen, S., and Ouzounis, C.A. (2002). An efficient algorithm for large-scale detection of protein families. Nucleic Acids Res *30*, 1575-1584.

Falquet, L., Pagni, M., Bucher, P., Hulo, N., Sigrist, C.J.A., Hofmann, K., and Bairoch, A. (2002). The PROSITE database, its status in 2002. Nucleic Acids Research *30*, 235-238.

Griffiths-Jones, S., Moxon, S., Marshall, M., Khanna, A., Eddy, S.R., and Bateman, A. (2005). Rfam: annotating non-coding RNAs in complete genomes. Nucleic Acids Research *33*, D121-D124.

Haas, B.J., Delcher, A.L., Mount, S.M., Wortman, J.R., Smith, R.K., Jr., Hannick, L.I., Maiti, R., Ronning, C.M., Rusch, D.B., Town, C.D.*, et al.* (2003). Improving the Arabidopsis genome annotation using maximal transcript alignment assemblies. Nucleic Acids Res *31*, 5654-5666.

Haas, B.J., Papanicolaou, A., Yassour, M., Grabherr, M., Blood, P.D., Bowden, J., Couger, M.B., Eccles, D., Li, B., Lieber, M.*, et al.* (2013). De novo transcript sequence reconstruction from RNA-seq using the Trinity platform for reference generation and analysis. Nature protocols *8*, 1494-1512.

Haas, B.J., Salzberg, S.L., Zhu, W., Pertea, M., Allen, J.E., Orvis, J., White, O., Buell, C.R., and Wortman, J.R. (2008). Automated eukaryotic gene structure annotation using EVidenceModeler and the program to assemble spliced alignments. Genome Biology *9*.

Haft, D.H., Selengut, J.D., and White, O. (2003). The TIGRFAMs database of protein families. Nucleic Acids Research *31*, 371-373.

Kanehisa, M., Furumichi, M., Tanabe, M., Sato, Y., and Morishima, K. (2017). KEGG: new perspectives on genomes, pathways, diseases and drugs. Nucleic Acids Res *45*, D353-D361.

Katoh, K., Rozewicki, J., and Yamada, K.D. (2019). MAFFT online service: multiple sequence alignment, interactive sequence choice and visualization. Brief Bioinform *20*, 1160-1166.

Kent, W.J. (2002). BLAT - The BLAST-like alignment tool. Genome Research *12*, 656-664.

Kimmel, C.B., Ballard, W.W., Kimmel, S.R., Ullmann, B., and Schilling, T.F. (1995). Stages of embryonic-development of the zebrafish. Developmental Dynamics *203*, 253-310.

Korf, I. (2004). Gene finding in novel genomes. BMC Bioinformatics *5*, 1-9.

Kumar, P., Henikoff, S., and Ng, P.C. (2009). Predicting the effects of coding non-synonymous variants

on protein function using the SIFT algorithm. Nature protocols *4*, 1073-1081.

Kuraku, S., Zmasek, C.M., Nishimura, O., and Katoh, K. (2013). aLeaves facilitates on-demand exploration of metazoan gene family trees on MAFFT sequence alignment server with enhanced interactivity. Nucleic Acids Res *41*, W22-28.

Larkin, M.A., Blackshields, G., Brown, N.P., Chenna, R., McGettigan, P.A., McWilliam, H., Valentin, F., Wallace, I.M., Wilm, A., Lopez, R.*, et al.* (2007). Clustal W and clustal X version 2.0. Bioinformatics *23*, 2947-2948.

Lees, J.G., Lee, D., Studer, R.A., Dawson, N.L., Sillitoe, I., Das, S., Yeats, C., Dessailly, B.H., Rentzsch, R., and Orengo, C.A. (2014). Gene3D: Multi-domain annotations for protein sequence and comparative genome analysis. Nucleic Acids Research *42*, D240-D245.

Li, H., and Durbin, R. (2010). Fast and accurate long-read alignment with Burrows-Wheeler transform. Bioinformatics *26*, 589-595.

Li, L., Stoeckert, C.J., and Roos, D.S. (2003). OrthoMCL: Identification of ortholog groups for eukaryotic genomes. Genome Research *13*, 2178-2189.

Liu, B., Shi, Y., Yuan, J., Hu, X., Zhang, H., Li, N., Li, Z., Chen, Y., Mu, D., and Fan, W. (2013). Estimation of genomic characteristics by analyzing k-mer frequency in de novo genome projects. arXiv preprint arXiv:13082012.

Lowe, T.M., and Eddy, S.R. (1997). tRNAscan-SE: A program for improved detection of transfer RNA genes in genomic sequence. Nucleic Acids Research *25*, 955-964.

Lupas, A., Vandyke, M., and Stock, J. (1991). Predicting coiled coils from protein sequences. Science *252*, 1162-1164.

Majoros, W.H., Pertea, M., and Salzberg, S.L. (2004). TigrScan and GlimmerHMM: two open source

ab initio eukaryotic gene-finders. Bioinformatics *20*, 2878-2879.

Murrell, B., Weaver, S., Smith, M.D., Wertheim, J.O., Murrell, S., Aylward, A., Eren, K., Pollner, T., Martin, D.P., Smith, D.M.*, et al.* (2015). Gene-Wide Identification of Episodic Selection. Molecular biology and evolution *32*, 1365-1371.

Nawrocki, E.P., and Eddy, S.R. (2013). Infernal 1.1: 100-fold faster RNA homology searches. Bioinformatics *29*, 2933-2935.

O'Leary, N.A., Wright, M.W., Brister, J.R., Ciufo, S., Haddad, D., McVeigh, R., Rajput, B., Robbertse, B., Smith-White, B., Ako-Adjei, D.*, et al.* (2016). Reference sequence (RefSeq) database at NCBI: current status, taxonomic expansion, and functional annotation. Nucleic Acids Res *44*, D733-745.

Parra, G., Bradnam, K., and Korf, I. (2007). CEGMA: a pipeline to accurately annotate core genes in eukaryotic genomes. Bioinformatics *23*, 1061-1067.

Patel, R.K., Mukesh, J., and Zhanjiang, L. (2012). NGS QC Toolkit: A Toolkit for Quality Control of Next Generation Sequencing Data. PloS one *7*, e30619-.

Pond, S.L.K., Frost, S.D.W., and Muse, S.V. (2005). HyPhy: hypothesis testing using phylogenies. Bioinformatics *21*, 676-679.

Priyam, A., Woodcroft, B.J., Rai, V., Munagala, A., Moghul, I., Ter, F., Gibbins, M.A., Moon, H., Leonard, G., and Rumpf, W. (2015). Sequenceserver: a modern graphical user interface for custom BLAST databases. Biorxiv, 033142.

Punta, M., Coggill, P.C., Eberhardt, R.Y., Mistry, J., Tate, J., Boursnell, C., Pang, N., Forslund, K., Ceric, G., Clements, J.*, et al.* (2012). The Pfam protein families database. Nucleic Acids Research *40*, D290-D301.

Robinson, M.D., and Oshlack, A. (2010). A scaling normalization method for differential expression

analysis of RNA-seq data. Genome Biol *11*, R25.

Schartl, M., Walter, R.B., Shen, Y., Garcia, T., Catchen, J., Amores, A., Braasch, I., Chalopin, D., Volff, J.N., Lesch, K.P.*, et al.* (2013). The genome of the platyfish, Xiphophorus maculatus, provides insights into evolutionary adaptation and several complex traits. Nat Genet *45*, 567-572.

Schultz, J., Milpetz, F., Bork, P., and Ponting, C.P. (1998). SMART, a simple modular architecture research tool: Identification of signaling domains. Proceedings of the National Academy of Sciences of the United States of America *95*, 5857-5864.

Seppey, M., Manni, M., and Zdobnov, E.M. (2019). BUSCO: Assessing Genome Assembly and Annotation Completeness. Methods Mol Biol *1962*, 227-245.

Simao, F.A., Waterhouse, R.M., Ioannidis, P., Kriventseva, E.V., and Zdobnov, E.M. (2015). BUSCO: assessing genome assembly and annotation completeness with single-copy orthologs. Bioinformatics *31*, 3210-3212.

Solovyev, V., Kosarev, P., Seledsov, I., and Vorobyev, D. (2006). Automatic annotation of eukaryotic genes, pseudogenes and promoters. Genome Biology *7*.

Spielman, S.J., Weaver, S., Shank, S.D., Magalis, B.R., Li, M., and Kosakovsky Pond, S.L. (2019). Evolution of Viral Genomes: Interplay Between Selection, Recombination, and Other Forces. Methods Mol Biol *1910*, 427-468.

Stamatakis, A. (2014). RAxML version 8: a tool for phylogenetic analysis and post-analysis of large phylogenies. Bioinformatics *30*, 1312-1313.

Stanke, M., Keller, O., Gunduz, I., Hayes, A., Waack, S., and Morgenstern, B. (2006). AUGUSTUS: ab initio prediction of alternative transcripts. Nucleic Acids Research *34*, W435-W439.

Tamura, K., Stecher, G., Peterson, D., Filipski, A., and Kumar, S. (2013). MEGA6: Molecular

Evolutionary Genetics Analysis Version 6.0. Molecular biology and evolution *30*, 2725-2729.

Tang, H., and Thomas, P.D. (2016). PANTHER-PSEP: predicting disease-causing genetic variants using position-specific evolutionary preservation. Bioinformatics *32*, 2230-2232.

Tania, L., H., A.A., Elisabeth, C., Guillaume, K., Karine, M., Catherine, R., Virginie, B., Edouard, d.C., Corinne, L., and Delphine, B. (2009). HAMAP: a database of completely sequenced microbial proteome sets and manually curated microbial protein families in UniProtKB/Swiss-Prot. Nucleic Acids Research, 471-478.

Tarailo-Graovac, M., and Chen, N. (2009). Using RepeatMasker to identify repetitive elements in genomic sequences. Curr Protoc Bioinformatics *Chapter 4*, Unit 4 10.

The UniProt, C. (2017). UniProt: the universal protein knowledgebase. Nucleic Acids Res *45*, D158-D169.

Thomas, P.D., Campbell, M.J., Kejariwal, A., Mi, H.Y., Karlak, B., Daverman, R., Diemer, K., Muruganujan, A., and Narechania, A. (2003). PANTHER: A library of protein families and subfamilies indexed by function. Genome Research *13*, 2129-2141.

Trapnell, C., Pachter, L., and Salzberg, S.L. (2009). TopHat: discovering splice junctions with RNA-Seq. Bioinformatics *25*, 1105-1111.

Trapnell, C., Roberts, A., Goff, L., Pertea, G., Kim, D., Kelley, D.R., Pimentel, H., Salzberg, S.L., Rinn, J.L., and Pachter, L. (2014). Differential gene and transcript expression analysis of RNA-seq experiments with TopHat and Cufflinks (vol 7, pg 562, 2012). Nature protocols *9*, 2513-2513.

Venkatesh, B., Lee, A.P., Ravi, V., Maurya, A.K., Lian, M.M., Swann, J.B., Ohta, Y., Flajnik, M.F., Sutoh, Y., Kasahara, M.*, et al.* (2014). Elephant shark genome provides unique insights into gnathostome evolution. Nature *505*, 174-179.

Walker, B.J., Abeel, T., Shea, T., Priest, M., Abouelliel, A., Sakthikumar, S., Cuomo, C.A., Zeng, Q.D., Wortman, J., Young, S.K*., et al.* (2014). Pilon: An Integrated Tool for Comprehensive Microbial Variant Detection and Genome Assembly Improvement. PloS one *9*.

Ward, R., Zemlak, T., Innes, B., Last, P., and Hebert, P. (2005). DNA barcoding Australia s fish species. *360*, 1847-1857.

Waterhouse, R.M., Seppey, M., Simao, F.A., Manni, M., Ioannidis, P., Klioutchnikov, G., Kriventseva, E.V., and Zdobnov, E.M. (2017). BUSCO applications from quality assessments to gene prediction and phylogenomics. Molecular biology and evolution.

Wu, C.H., Nikolskaya, A., Huang, H.Z., Yeh, L.S.L., Natale, D.A., Vinayaka, C.R., Hu, Z.Z., Mazumder, R., Kumar, S., Kourtesis, P*., et al.* (2004). PIRSF: family classification system at the Protein Information Resource. Nucleic Acids Research *32*, D112-D114.

Yang, J., Chen, X., Bai, J., Fang, D., Qiu, Y., Jiang, W., Yuan, H., Bian, C., Lu, J., He, S*., et al.* (2016). The Sinocyclocheilus cavefish genome provides insights into cave adaptation. BMC Biol *14*, 1.

Yang, Z.H. (2007). PAML 4: Phylogenetic analysis by maximum likelihood. Molecular biology and evolution *24*, 1586-1591.

Zdobnov, E.M., and Apweiler, R. (2001). InterProScan - an integration platform for the signature-recognition methods in InterPro. Bioinformatics *17*, 847-848.

Zheng, G.X., Lau, B.T., Schnall-Levin, M., Jarosz, M., Bell, J.M., Hindson, C.M., Kyriazopoulou-Panagiotopoulou, S., Masquelier, D.A., Merrill, L., Terry, J.M*., et al.* (2016). Haplotyping germline and cancer genomes with high-throughput linked-read sequencing. Nat Biotechnol *34*, 303-311.
